# Direct and simultaneous observation of ultrafast electron and hole dynamics in germanium

**DOI:** 10.1038/ncomms15734

**Published:** 2017-06-01

**Authors:** Michael Zürch, Hung-Tzu Chang, Lauren J. Borja, Peter M. Kraus, Scott K. Cushing, Andrey Gandman, Christopher J. Kaplan, Myoung Hwan Oh, James S. Prell, David Prendergast, Chaitanya D. Pemmaraju, Daniel M. Neumark, Stephen R. Leone

**Affiliations:** 1 Department of Chemistry, University of California at Berkeley, Berkeley, California 94720, USA; 2 Materials Sciences Division, Lawrence Berkeley National Laboratory, Berkeley, California 94720, USA; 3 The Molecular Foundry, Lawrence Berkeley National Laboratory, Berkeley, California 94720, USA; 4 Theory Institute for Materials and Energy Spectroscopies, SLAC National Accelerator Laboratory, Menlo Park, California 94025, USA; 5 Chemical Sciences Division, Lawrence Berkeley National Laboratory, Berkeley, California 94720, USA; 6 Department of Physics, University of California, Berkeley, California 94720, USA

## Abstract

Understanding excited carrier dynamics in semiconductors is crucial for the development of photovoltaics and efficient photonic devices. However, overlapping spectral features in optical pump-probe spectroscopy often render assignments of separate electron and hole carrier dynamics ambiguous. Here, ultrafast electron and hole dynamics in germanium nanocrystalline thin films are directly and simultaneously observed by ultrafast transient absorption spectroscopy in the extreme ultraviolet at the germanium M_4,5_ edge. We decompose the spectra into contributions of electronic state blocking and photo-induced band shifts at a carrier density of 8 × 10^20^ cm^−3^. Separate electron and hole relaxation times are observed as a function of hot carrier energies. A first-order electron and hole decay of ∼1 ps suggests a Shockley–Read–Hall recombination mechanism. The simultaneous observation of electrons and holes with extreme ultraviolet transient absorption spectroscopy paves the way for investigating few- to sub-femtosecond dynamics of both holes and electrons in complex semiconductor materials and across junctions.

## Abstract (web summary)

Understanding excited carrier dynamics in semiconductors is central to the continued development of optoelectronic devices. Using extreme ultraviolet transient absorption spectroscopy, Zürch *et al*. directly and simultaneously observe ultrafast electron and hole dynamics in germanium thin films.

Investigation of the ultrafast photoexcited electronic response in semiconductors has provided invaluable insights into the carrier dynamics and dielectric properties of many materials. Germanium and its alloys with Si have promise for creating mid-infrared optoelectronics^1^ and multijunction solar cells with higher efficiency^2^. However, the multiple energetically similar valleys complicate understanding of carrier thermalization and population inversion^3^ following photoexcitation. A technique is clearly needed that can monitor carrier dynamics of both electrons and holes in the relevant valleys with few-femtosecond time resolution. With the advent of femtosecond laser techniques, electron and hole scattering in semiconductors has been observed individually and characterized by optical and infrared pump-probe experiments^4^,^5^. However, the time-resolved observation and characterization of electron and hole kinetics simultaneously in the optical regime is challenging due to overlapping spectral signatures requiring narrowband excitation to separate pump from probe, which inherently limits the temporal resolution.

Ultrafast transient absorption spectroscopy in the extreme ultraviolet (XUV) is an important technique for studying electron dynamics at the sub-femtosecond and few-femtosecond timescale^6^,^7^. This technique has been successfully applied to investigate the dielectric response of insulators^8^,^9^ and carrier dynamics in semiconductors^10^,^11^ down to sub-femtosecond timescales. In ultrafast XUV transient absorption spectroscopy, a visible-to-near infrared (VIS-NIR) pump pulse excites carriers first, and after a given time delay *τ*, a broadband pulse in the XUV consisting of an isolated attosecond pulse or a short attosecond pulse train generated by high harmonic generation^12^ excites core-level electrons into the valence and conduction bands (CBs). The changes in electronic population in the valence and CBs lead to changes in XUV absorption from the core-level excitations, which we refer to as electronic state blocking (SB). In addition, the energy shift of valence and CBs induced by electron–hole excitations will also be recorded in XUV transient absorption. The core-level excitation with XUV photons is element specific, making the technique advantageous for investigating carrier dynamics in heteroatomic, ternary and quaternary systems. The photon energy of the broadband XUV pulse lies far above the interband excitation energies, and therefore this technique in principle allows simultaneous capture of electron and hole dynamics without further inducing interband carrier excitations.

Germanium is a group IV semiconductor with an indirect bandgap of 0.66 eV. Previous optical experiments observed electron scattering from one part of the band structure to another, that is, intervalley scattering, occurring on a few hundreds of femtosecond timescale^13^,^14^,^15^,^16^. In separate experiments, interband scattering of holes has been investigated^17^,^18^. Recently, studies of the intervalley scattering of carriers in germanium have attracted renewed interest due to the prospect of spin polarization induced in the scattering event, which is useful for the development of spintronics^18^,^19^,^20^,^21^.

Here, we address the need for measuring carrier dynamics more comprehensively by using ultrafast transient absorption in the XUV region of the spectrum, with specific application of the method to electron and hole dynamics in photoexcited germanium. A model is established that allows decomposing the measured raw transient absorption data into contributions of SB, shifts of the excited state spectrum and broadening. We show how the carrier dynamics can be extracted by separating spin-orbit components in the XUV. Following this, XUV transient absorption at the germanium M_4,5_ edge can spectrally resolve signatures of electrons in the CB and holes in the valence band (VB) simultaneously, which enables the time- and energy-resolved tracking of electron and hole kinetics. Carrier-induced and heat-induced band shifts are observed in addition to state filling, synthesizing a complete picture of the photoinduced dynamics. The electron and hole relaxation and recombination times within a single experiment on nanocrystalline germanium thin films are successfully characterized.

## Results

### XUV transient absorption experiment on germanium

**Figure 1:**
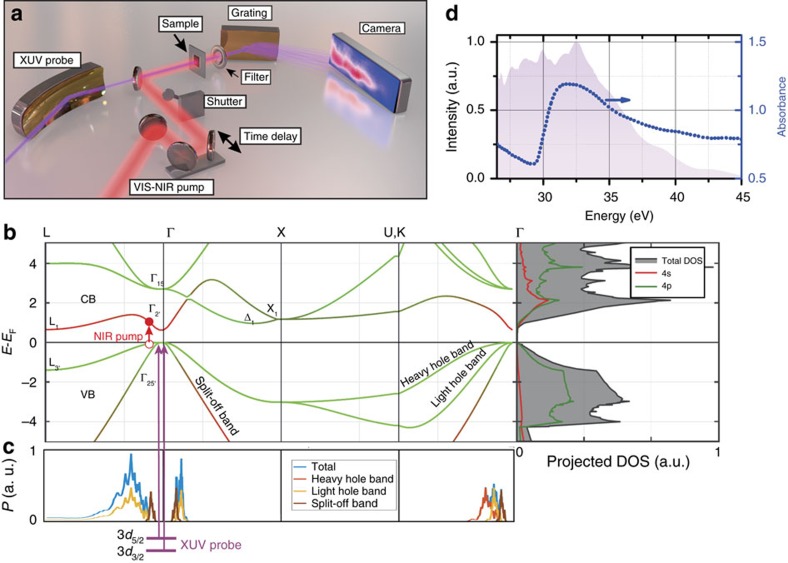
XUV ultrafast transient absorption spectroscopy in germanium. (**a**) In the experiment a time-delayed broadband XUV pulse is used to probe the transient absorption of a nanocrystalline germanium thin film after excitation with a broadband VIS-NIR pump pulse. (**b**) Band diagram and projected density of states (DOS) for germanium, calculated here by density functional theory calculation (see Methods section). The VIS-NIR pump pulse initially promotes electrons (filled red circle) into the CB leaving behind a hole (open red circle) in the VB. (**c**) Transition probability *P* for the specific VIS-NIR pump pulse used in this experiment at different parts of the band diagram (see Supplementary Note 2). The large bandwidth of the pump pulse allows to generate holes at all slopes of the Γ valley in the light-hole band (yellow solid line in **c**) and heavy-hole band (red solid line in **c**) as well as the split-off band (brown solid line in **c**) by one-photon transitions without the assistance of phonons (that is, direct transitions). At the M_4,5_ edge the XUV pulse probes the transient state in the VB and CB from a 3*d* core level as indicated by the purple lines and arrows in **b**,**c**; the 3*d* core level has a significant spin-orbit splitting in germanium of 0.58 eV (ref. 23), and transitions from both spin-orbit states are observed to those parts of the bands that are of 4*p* orbital character. The VB and CB in germanium are primarily of 4*s* and 4*p* orbital character. The orbital character is encoded by a red and green colour code, respectively, for 4*s* and 4*p* orbital character in **b**. (**d**) The spectrum of the broadband XUV probe pulse covers the M_4,5_ edge of germanium (see absorbance in dotted blue line in **d**) around 30 eV.

 In Fig. 1 the XUV transient absorption experiment performed on a nanocrystalline germanium film is illustrated schematically. In the experimental apparatus (Fig. 1a), a time-delayed VIS-NIR pump pulse is collinearly superimposed with a broadband XUV pulse and focused onto the sample. The transient absorption signal Δ*A*_meas_(*E*, *τ*)=*A*_p_(*E*, *τ*)−*A*(*E*) is defined by the difference between the absorbance of the excited (pumped) thin film *A*_p_ and the static absorbance *A*. Depending on the time delay *τ* between the VIS-NIR excitation (pump) pulse and XUV probe pulse, the transient absorption as a function of wavelength and time is recorded by spectrally resolving the XUV light after the sample using a spectrometer. A detailed explanation of the experimental apparatus and sample characterization is in the Methods section and Supplementary Note 1. The samples are produced by electron beam deposition and annealing, leading to nanocrystalline domains, most likely containing significant defects. The few-cycle VIS-NIR pulse spanning 1.2–2.5 eV photon energy promotes electrons across the bandgap from the VB into the CB (Fig. 1b). Given the spectrum of the exciting VIS-NIR pulse, the region of possible carrier excitation within the Brillouin zone of germanium is calculated (see Supplementary Note 2). Figure 1c summarizes the possible optical excitations from the heavy-hole, light-hole and split-off bands to the four low-lying CBs. All significant photon energies contained in the VIS-NIR pulse allow for a direct one-photon transition between the bands, hence indirect transitions assisted by phonons are expected to play a minor role for the pump pulse used^22^. The excitation pulse mainly excites carriers within the Γ valley and the XUV transient absorption tracks the intervalley and intravalley scattering, that is, the thermalization of holes and electrons, and ultimately the recombination of the carriers. The broadband XUV pulse, whose spectrum spans the germanium M_4,5_ absorption edge (Fig. 1d), allows to probe an electron originating from a germanium 3*d* core level to a respective VB or CB state. In general, the XUV pulse can probe the transient populations in both the CB and VB as both are of partial 4*p* orbital character (green colour code in Fig. 1b), while transitions to parts of the bands that are of 4*s* character are forbidden by dipole selection rules (red colour code in Fig. 1b) and invisible to the probe. The instrumental response time has an upper bound estimated to be ∼6 fs (see Methods section).

### Decomposition of transient absorption spectra

**Figure 2:**
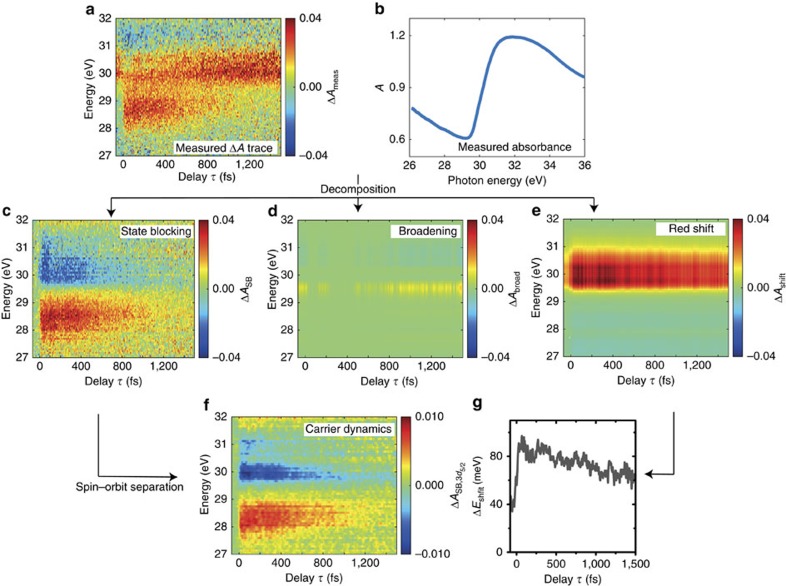
Decomposition of the contributions from SB and broadening and band shifts. (**a**) Raw transient absorption data. Using the measured static absorbance (**b**) the measured Δ*A*_meas_ trace (**a**) is decomposed into three major components: (**c**) SB, (**d**) broadening of the excited state and (**e**) a redshift of the ground state, via an iterative algorithm, see text for details. (**f**) Modifying the SB contribution with a subsequent spin-orbit separation allows quantitative visualization of the electron and hole contributions, referred to as carrier dynamics. (**g**) The amount of redshift Δ*E*_shift_(*τ*) over the delay shows a constant non-zero shift for negative time delays, which is heat induced from previous laser pulses, and a time dependence for positive delays.

Raw transient absorption spectra Δ*A*_meas_(*E*,*τ*) at different time delays *τ* are shown in Fig. 2a. The sample was illuminated using a VIS-NIR pulse with an intensity of 2.0 × 10^11^ W cm^−2^, exciting ∼8 × 10^20^ carriers per cubic centimetre or ∼0.3% of the total number density (see Methods section). This constitutes a high carrier density regime where carrier–carrier scattering effects are expected to play a role. In the time delay span measured from −50 fs to 1.5 ps, where negative delay means the XUV pulse arrives first, transient signals are observed for all time delays. Several features with positive and negative signs in Δ*A*_meas_ are observed in the transient absorption signal, with most of the observable dynamics taking place during the first picosecond following excitation. Overlapping spectral features arise from the spin-orbit splitting of the 3*d*_5/2_ and 3*d*_3/2_ core-level transitions; this splitting, 0.58 eV, is comparable to the bandgap (0.66 eV)^23^. One expects to observe dynamical features of band shifts due to the excited electron–hole plasma^24^ and phonon heating of the lattice^25^,^26^, carrier excitation-induced core-level shifts and spectral broadening of the M_4,5_ edge (Fig. 2b)^27^, and electronic SB from excited carriers. As a whole, the complexity of underlying effects that contribute to the observed transient absorption spectra require disentangling these from the raw data to study the contributions individually.

We introduce an iterative procedure that accounts for possible spectral broadening, shifting and SB, and seeks to break up these individual contributions from the measured raw data. In this procedure, the individual contributions by shifting and broadening can be calculated from the measured static absorbance *A*(*E*) (Fig. 2b) of the sample using a linear energy shift Δ*E*_shift_ and by convoluting the absorbance with a Gaussian of width *σ*, respectively. The SB can be estimated in the first iteration by two Gaussians having a width corresponding to the bandwidth of the VIS-NIR pulse with opposite signs for electrons and holes spaced by the central energy of the VIS-NIR pulse and subsequently by considering the spin-orbit splitting of the core level. The assumption of a Gaussian shape of the SB is chosen as reasonable compromise between the expected initial distribution related to the NIR-VIS spectrum, which transitions into a Fermi-Dirac-type distribution after rapid thermalization, convoluted with the instrumental resolution of the instrument. The time dependence of the SB is first estimated as a single exponential decay with a characteristic time constant retrieved from the experimental data. By minimizing an error metric taking the sum of these components and the measured signal into account, in the first iteration the underlying time-dependent shift Δ*E*_shift_(*τ*) and broadening *σ*(*τ*) are estimated. As a convention, here a positive value of Δ*E*_shift_(*τ*) means a redshift of the excited state spectrum. In subsequent iterations, the SB component Δ*A*_SB_(*E*, *τ*) (Fig. 2c) is refined along with refinement of Δ*E*_shift_(*τ*) and *σ*(*τ*), converging after five iterations. It is found that broadening Δ*A*_broad_(*E*,*τ*) plays only a minor role in the experiments presented here and constitutes signals at the noise level (Fig. 2d). The major contributions arise from a time-dependent redshift Δ*A*_shift_(*E*,*τ*) (Fig. 2e) and the SB Δ*A*_SB_(*E*,*τ*) (Fig. 2c) following carrier excitation. A detailed description of the iterative procedure is given in Supplementary Note 3.

As noted, the SB transient obtained (Fig. 2c) contains contributions from both 3*d*_5/2_ and 3*d*_3/2_ core-level transitions. A Fourier reconstruction method (see Methods section and Supplementary Note 4) is applied to the transient absorption signal to retrieve the contribution from a single spin-orbit state transition (3*d*_5/2_). Figure 2f shows the spin-orbit-separated SB transient 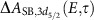 that we will subsequently refer to as carrier dynamics.

In the time-dependent band shift Δ*E*_shift_(*τ*) (Fig. 2g) at negative time delays, a constant redshift is measured that originates from a heat-induced band shift owing to the thin film being heated up in the multipulse exposure experiment. This transient feature allows retrieving the temperature of the thin film, which ranges from 325 to 475 K for the data presented here (see Methods section and Supplementary Note 5).

### Feature assignment in experimental transient absorption data

**Figure 3:**
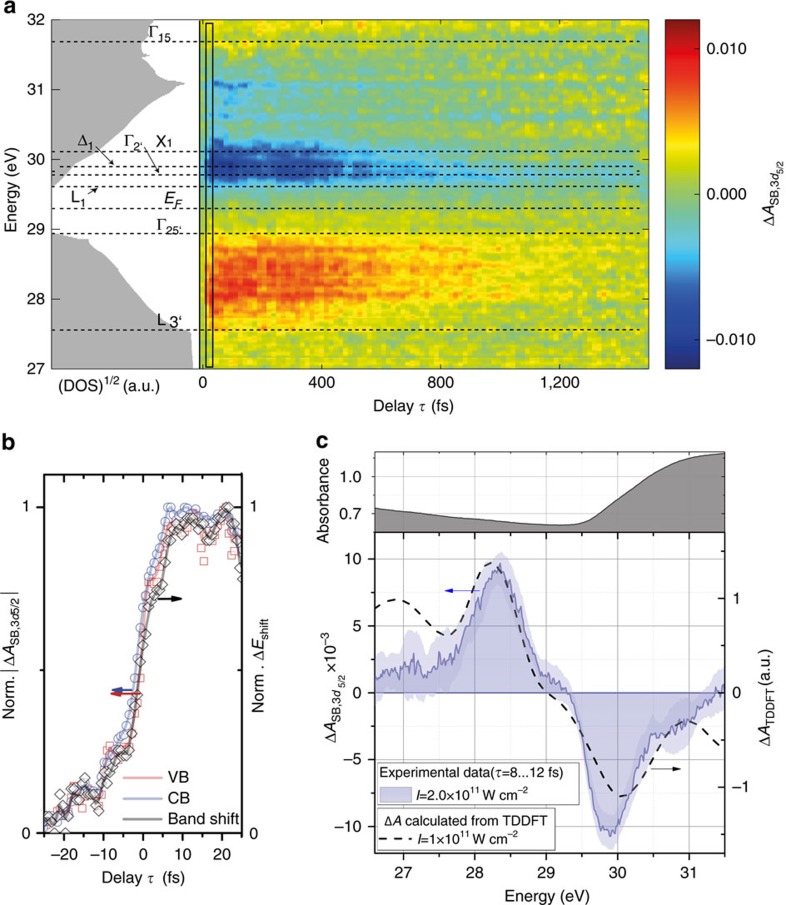
Differential absorption experiment in comparison to first-principles calculations. (**a**) A carrier dynamics signal 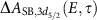 (*I*=2 × 10^11^  W cm^−2^) features positive and negative differential absorption in the VB and CB, respectively. Positive time delays correspond to the VIS-NIR pump pulse arriving before the XUV probe pulse. Comparison with a calculated density of states (DOS) allows assigning characteristic valleys of the band structure to the measured energy axis (cf. Fig. 1c). (**b**) The absolute values of the rises of the two main transient features around 28.3 eV (VB) and 29.9 eV (CB) are associated with electrons (blue open circles) and holes (red open squares), respectively, exhibiting a rise time limited by the duration of the VIS-NIR pulse. The measured band shift Δ*E*_shift_(*τ*) (black open diamonds) also follows the carrier excitation within the instrumental response time. The solid lines in **b** are moving averages to guide the eye. In (**c**) the differential absorption of the carrier dynamics directly after VB to CB excitation, that is, for positive time delays averaged over *τ*=8 to 12 fs (indicated by the black rectangle in **a**), is shown. The shaded error bar in (**c**) corresponds to the s.d. of individual data points within the averaging window. The dashed black line shows the differential absorption calculated from a TDDFT calculation assuming pulses with a peak intensity of 10^11^ W cm^−^^2^.

Figure 3a shows the retrieved carrier dynamics 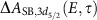 (same as Fig. 2f) with assignments of the valleys in germanium (cf. Fig. 1b). In general, two features are apparent, a broad positive feature around 28.2 eV and a broad negative feature spanning 29.5 to 31.0 eV. The signs of the carrier dynamics features can be understood as follows. With VIS-NIR excitation of electrons from VB to CB, holes are created in the VB, resulting in an increase of XUV absorption below the M_4,5_ edge. Oppositely, the VIS-NIR-excited electrons in the CB block XUV transitions from the core level, leading to a decrease in XUV absorption, and, thus, a negative Δ*A*. The carrier dynamics features as well as the band shift Δ*E*_shift_(*τ*) rise within the instrumental response time (Fig. 3b) closely following the exciting VIS-NIR pulse. The slow rise of the signal before time zero can be associated with a small pedestal or prepulse, for which the allowed one-photon transition in this experiment is susceptible to observation, in contrast to previous semiconductor attosecond experiments that used nonlinear excitation schemes^10^,^11^.

To support the assignment of the features in the carrier dynamics signal, the profiles averaged over time delays between +8 to +12 fs (black rectangle in Fig. 3a) are compared to differential absorption profiles obtained by first-principles calculations (Fig. 3c). Here, real-time time-dependent density functional theory (TDDFT) was first employed to calculate the electronic excitation of crystalline germanium by a VIS-NIR pulse comparable to the one used in the experiments (see Methods section). From the retrieved populations in the CB and VB following excitation, an excited-state X-ray absorption spectrum (XAS) was then calculated to obtain a transient absorption spectrum (dashed lines in Fig. 3c) with respect to a calculated XAS ground state spectrum, where both spectra are calculated for a single spin-orbit state. The comparison of the calculated (dashes in Fig. 3c) and experimental transient absorption (blue solid area in Fig. 3c) strongly corroborates the assignment of electrons and holes to the negative and positive carrier dynamics feature, respectively. This assignment is further supported by the binding energy of the 3*d*_5/2_ core level at 29.2 eV coinciding with the gap between the two main features relating to the bandgap. The deviations at higher electron and hole energies in Fig. 3c can be explained by the TDDFT calculation underestimating electron–hole interaction effects and neglecting carrier–phonon scattering, carrier–carrier scattering and relaxation processes, thus overestimating state filling at higher energies. In the experiment, carriers can immediately scatter, assisted by phonons or other carriers, to regions in **k** space not accessible by direct optical transitions and hence on average leave more transitions open at a given energy, thus mitigating band-filling effects.

### Electron kinetics

**Figure 4:**
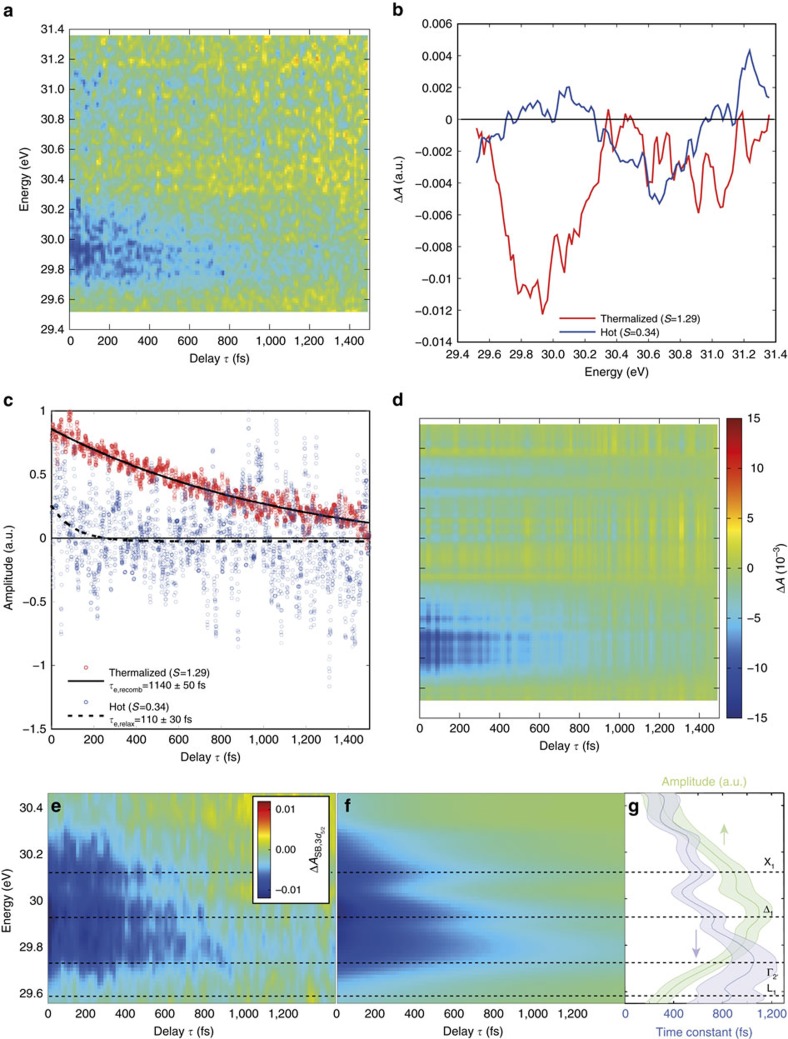
Electron kinetics in nanocrystalline germanium following ultrafast excitation. (**a**) The measured change of absorbance in the carrier dynamics in the spectral region of the CB decomposed into two singular value components. (**b**,**c**) The spectrum and the time dynamics of these singular value components, respectively. (**d**) Calculating a transient signal from both components and their time dependence yields good agreement with the measurement. (**a**,**d**) Share the colour bar indicated in **d**. The temporal dependence of the stronger component can be best described by a single exponential decay with a time constant of Δ*τ*_e,recomb_=(1,140±50) fs associated with the carrier recombination. The weaker component has a time constant of *τ*_e,relax_=(110±30) fs, suggesting a fast relaxation of hot electrons from higher energies to lower energies. (**e**) For a section of the CB near the band edge, a single exponential decay is fit to each energy slice of the data, which allows the construction of a map of the dynamics of hot electrons versus energy (**f**), which is in reasonable agreement with the data. (**g**) The obtained time constants versus energy (blue line with shaded error band (**g**)) indicate increased lifetimes of carriers at the CB valleys at several energies, with the longest lifetimes at the low-energy valleys, consistent with the SVD analysis. The shaded error bands correspond to the uncertainty of the retrieved fit parameters. See text discussion for further explanation and interpretation.

The carrier dynamics 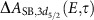 associated with the electrons (Fig. 4a) are analysed by singular value decomposition (SVD) to separate the underlying signals into different independent contributions of electrons and holes and to trace out their temporal evolution; see Methods section for details. The transient signal can be described by two major components whose spectrum is depicted in Fig. 4b along with the time dependence depicted in Fig. 4c. Calculating a carrier dynamics transient from these two SVD components (Fig. 4d) yields good agreement with the measured carrier dynamics transient (Fig. 4a). The component with larger amplitude and with the spectral distribution peaking near the band edge (red data points in Fig. 4b,c) can be assigned to the thermalized electrons. It exhibits a time dependence that can be fit with a single exponential decay with a time constant of *τ*_e,recomb_=(1,140±50) fs, which is characteristic of the recombination of the carriers, since this is the timescale when the transient carrier dynamics signal vanishes (Fig. 4a). The error bars are derived from the fitting to the experimental data. A single exponential describes the observed time dependence best and hints at a trap-assisted recombination process^28^, which is expected to be dominant in defect-rich samples^29^. The second largest component (blue data points in Fig. 4b,c) consists of higher-energy contributions and lower-energy components with reversed sign. This component (blue data points and fit with dashed black line in Fig. 4c) decays with *τ*_e,relax_=(110±30) fs, reversing its sign at the long time limit. This component of the SVD can be interpreted as high-energy electrons (hot carriers) relaxing very rapidly from higher-energy states to fill available states at lower energies within this relaxation time.

In addition, single exponential functions were fitted to profiles along the time-delay axis at energies between 29.6 and 30.4 eV (Fig. 4e,f), with time-zero amplitudes and time constants as free parameters (Fig. 4g). The time-zero amplitudes relate to the initial distribution of electrons (green line with shaded error band in Fig. 4g) and show that initially a broad distribution of electrons is excited. The time constants (blue line with shaded error band in Fig. 4g) provide a global view of the energy-specific lifetimes. One observes generally an increasing lifetime of carriers closer to the CB edge. Further, the CB valleys such as X_1_, Δ_1_, Γ_2′_ and weakly L_1_ stand out with significantly longer lifetimes, because the carriers relax to particular valleys and accumulate there.

### Hole kinetics

**Figure 5:**
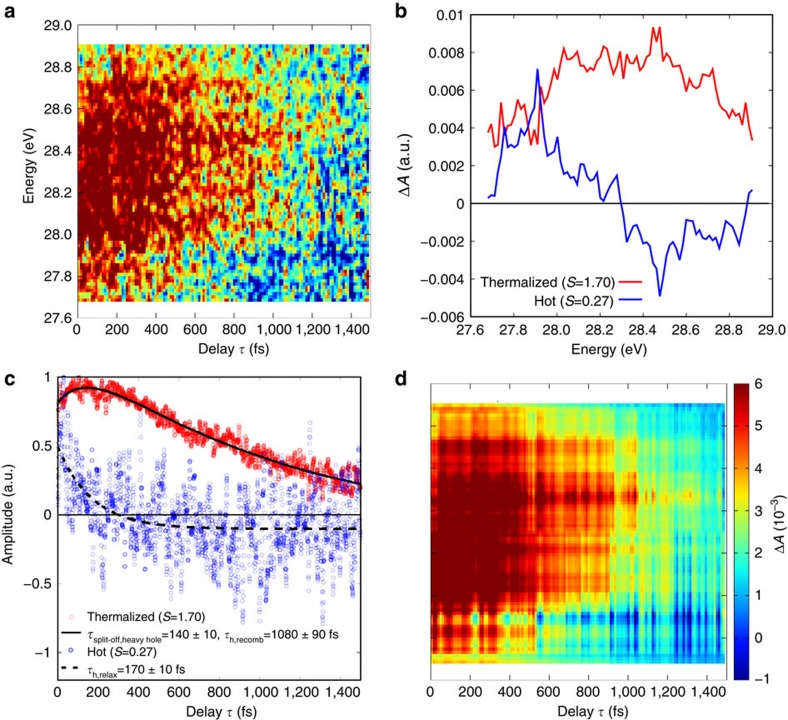
Hole kinetics in nanocrystalline germanium following ultrafast excitation. (**a**) The measured change of absorbance in the spectral region of holes can be decomposed into two singular value components. (**b**,**c**) The signal distribution and time dynamics of the two largest singular value components, respectively. (**d**) Their addition reproduces the observed transient absorption trace. (**a**,**d**) Share the colour bar indicated in **d**. The stronger component (red data in (**b**,**c**)) can be associated with thermalized holes. The growth of this component for *τ*_split-off,heavy hole_=(140±10) fs and subsequent decay in *τ*_h,recomb_=(1,080±90) fs can be understood by holes scattering from the split-off band to the heavy-hole band and subsequent recombination with electrons. The weaker component (blue data in (**b**,**c**)) can be associated with hot holes, which exhibit a fast relaxation to lower hole energies within *τ*_relax_=(170±10) fs. See text for further explanations.

Figure 5a shows the carrier dynamics signal that is associated with the holes, which is also analysed by SVD. The wide positive distribution at time zero spanning from 27.8 to 29.2 eV narrows down and decays as the time delay *τ* increases, representing the hot carrier relaxation and recombination of holes. Decomposing the transient absorption signal by SVD reveals two major components. For the largest component, an assignment is made to a thermalized hole distribution (Fig. 5b, red line), whose dynamics show first a rise, *τ*_split-off,heavy hole_= (140±10) fs, after the initial excitation and a subsequent *τ*_h,recomb_=(1,080±90) fs decay (Fig. 5c, red dots with solid black line) that are obtained by fitting a biexponential function. The assignment of the faster dynamics with the time constant *τ*_split-off,heavy hole_ to hole scattering pathways from the split-off band to heavy-hole band is based on the initial increase of the measured carrier concentrations. This is most clearly seen for time delays of 100–150 fs, where both the measured transients (Fig. 5a) and the thermalized component of the SVD (Fig. 5c) maximize. In Fig. 1c it is shown that excitation to the split-off band by the pump pulse, which is of partial 4*s* orbital character (Fig. 1b), is possible. Once carriers scatter from the split-off band to the heavy-hole band, which is predominantly of 4*p* orbital character, the carriers become observable to the XUV and increase the carrier dynamics signal. Since the VB is degenerate at the Γ point with predominantly 4*p* orbital character, another possible pathway would be hole relaxation within the split-off band towards the Γ point. However, this process has been previously found to be less efficient compared to the split-off band to heavy-hole band scattering process^17^. For other pathways such as carriers scattering from the heavy- or light-hole band into the split-off band, a reduction of amplitude, contrary to the observation, would be expected in the carrier dynamics signal.

The second largest component features a positive distribution at larger (hot) hole energies (∼28 eV) and a negative distribution near the band edge (Fig. 5b, blue line). The time evolution of this component starts at a positive value and becomes negative at the long time limit; it is fitted with a single exponential decay (Fig. 5c, blue dots with dashed black line) with a *τ*_relax_=(170±10) fs time constant. This indicates that carrier dynamics, albeit a signal proportional to the population distribution, changes sign at the long time limit. Multiplying the time dynamics and the carrier dynamics component together suggests that the second largest component represents a depletion of hot holes and an increase of low-energy holes as the singular vector in time decays from positive to negative value (Fig. 5c, blue dots and dashed black line), representing the hot hole relaxation.

### Time-dependent band shifts

The main contribution from the band shift observed here originates from the redshift of the CB, where the absorbance experiences a steep rise (inset Fig. 3c, upper panel). The VB region is attributed to energies where the absorbance is relatively flat, indicating that band shifts in either direction would result in weak contributions on the VB side. It is a feature that XUV transient absorption measurements capture differential quantities, where underlying small shifts can cause measurably broad signals if these changes occur in steep sections of the underlying ground state absorbance (cf. Fig. 2e). These detectable shifts can be significantly below the actual spectral resolution of the instrument.

**Figure 6:**
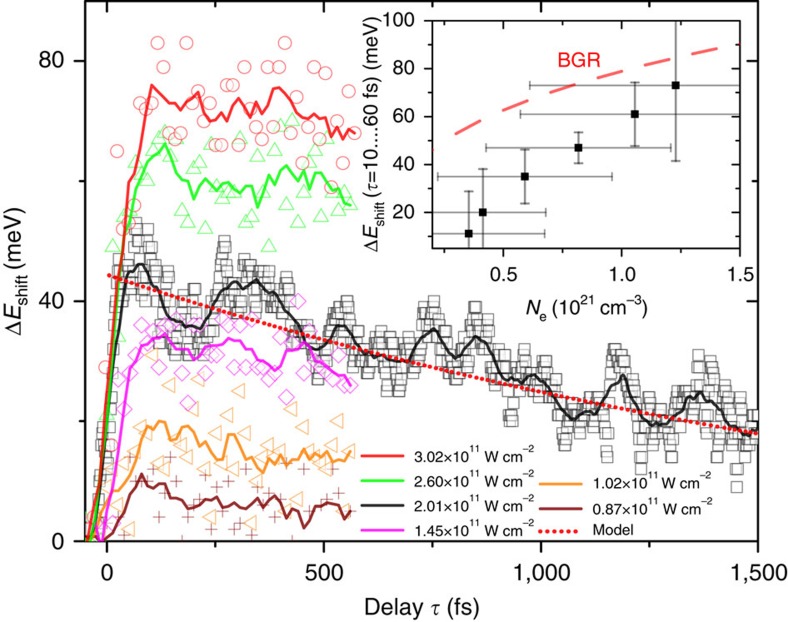
Band shift depending on time delay and intensity. Here the band shift Δ*E*_shift_=(*τ*) for different excitation intensities is depicted. The temporal behaviour suggests that the observed shift is predominantly due to a redshift of the CB due to carrier-induced band shift 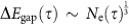, assuming a single exponential carrier decay with a time constant of 1.1 ps (red dotted line). In the inset the measured initial shifts (black squares) for different initial carrier densities is compared to an analytic calculation of the band shift. The calculated redshift of the CB (red dashed line, inset) due to bandgap renormalization (BGR) for different carrier densities is slightly larger than the measured band shifts. The vertical error bars in the inset correspond to the s.d. of the data points in the time delay segment. The horizontal error bars are derived by taking the uncertainties of the measured excitation fluence into account. See text for discussion.

In Figure 6, the heat-induced band shift has been subtracted to focus on the band shift following photoexcitation using different intensities. The carrier-induced band shift scales with the third root of the number of excited carriers 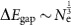 (see Supplementary Note 6) and is thus time dependent as the carrier density changes^24^, for example, by recombination. In the experiment, the observed redshift increases with increasing intensity and thus increased initial carrier density *N*_e_ in the CB. In Fig. 6 the time-dependent energy shift at one excitation intensity (black line in Fig. 6) is compared to 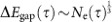 (red dotted line in Fig. 6) using a carrier density decay that follows an exponential with a time constant of 1.1 ps with increasing delay *τ*. The excellent agreement suggests that the observed band shift is predominantly caused by a carrier-induced dynamic redshift of the CB. The oscillations in the band shift signal may arise from phonon dynamics induced by carrier excitations. The phonon excitations lead to lattice strains that subsequently result in shifting of band energies. The impulsive and broadband excitation can cause excitation of overtones^30^ and multiphonon processes^31^, which can lead to a large bandwidth of excited phonon modes giving rise to the fast oscillations observed in the bandshift signal.

The excited carrier densities range from 0.4 × 10^21^ to 1.2 × 10^21^ cm^−3^ according to the TDDFT calculations using the pulse parameters of the experiment. In the inset of Fig. 6, the measured redshifts directly after excitation for the calculated carrier densities are compared to Δ*E*_gap_/2 (red dashed line, inset Fig. 6) to account for the fact that here only the redshift of the CB is observed. The overestimation of bandgap renormalization is due to the additional Burstein–Moss effect, which is a blueshifting of the absorption edges^32^,^33^ at carrier densities exceeding ∼10^19^ cm^−3^. In the X-ray absorption data this may also be counteracted by a core-level redshift due to the screening^10^, which is expected to be on the order of a few tens of meV. This, however, does not limit the extraction of the carrier dynamics contribution. An extended discussion can be found in Supplementary Note 6.

## Discussion

In the present experiment, electron and hole dynamics are studied simultaneously and with high time and energy resolution. The energetic separation of the electron and hole signals is of great value since band-related features and carrier-specific dynamics can be unambiguously assigned (Fig. 3a). The decoupling of the pump and probe spectral regions allows to excite samples with, for instance, ultrashort broadband laser pulses as in the present experiment to study ultrafast excitation dynamics and electronic relaxation processes of both carriers, or with longer monochromatic pulses for valley-specific excitation independent of the XUV probe pulse. The possibility for frequency-converting the ultrafast pump pulses will further extend the accessible materials. Converting the ultrashort pump pulse into the ultraviolet-to-blue spectral region^34^ will allow the investigation of wide-bandgap materials. Furthermore, thermalization dynamics such as the ∼110 fs relaxation time observed for the CB electrons as well as capturing the fast oscillations in the band shift that are likely related to coherent phonons and their overtones require the time resolution that ultrafast XUV transient absorption spectroscopy offers, which can be expanded towards attosecond transient absorption spectroscopy by isolating a single attosecond pulse from the attosecond pulse train.

Discussing the electron dynamics, the assignment of the CB valleys in Fig. 4e, is essential. Using energy-resolved line profiles and fitting single exponential decays at each energy for near-edge CB states, energy-resolved carrier lifetimes are extracted. The initial amplitudes of the exponential fits (green line with shaded error bars in Fig. 4g) suggests that initially a broad distribution of electrons is excited. Following excitation, the carriers undergo intervalley and intravalley scattering with phonons, effectively populating all bands within the energy range and relaxing the electrons to respective valleys. The measured time constant versus energy (blue line with shaded error bar in Fig. 4g) supports this intuitive picture. There, the time constant for higher-lying hot carrier states is the shortest, continually increasing towards the band edge. At the valleys, the measured time constants peak due to the carriers accumulating (see for example, energy assigned to X_1_ and Δ_1_ valleys in Fig. 4g). Hence, although the experiment averages over all **k** points in the Brillouin zone, the results suggest that characteristics of specific valleys can be extracted from the experiment due to the high DOS at the valleys, provided the involved valleys have sufficient energy spacing. At the germanium M_4,5_ edge this is the case, for example, for the X_1_ valley versus Γ_2′_, but not for L_1_ and Γ_2′_. Here, the achievable resolution is mainly limited by the core-hole lifetime broadening, which constrains the energy resolution to *δE*≈0.24 eV (ref. 23). For nanocrystalline samples, an additional broadening due to averaging over different crystal orientations is expected^35^. In the experiment, the slow time constants for decay in the Γ_2′_ and L_1_ valleys are on the order of 1.1 ps, which is consistent with the carrier decay time in the SVD analysis. The SVD components (Fig. 4b) can be understood as an ensemble average and the measured ∼100 fs relaxation time in the second SVD component corresponds to an average relaxation time that also includes electrons at higher energies, where the signal strength prohibits meaningful line profile measurements. It is worth noting that the weak amplitude in the L_1_ valley (Fig. 4g) is due to the high *s* orbital character of the states, which limits its visibility in the XUV spectrum. Further, Γ–L and X–L intervalley scattering occurs on a 200–300 fs timescale, which together with the observed lifetime of carriers at the CB edge gives rise to a low overall transient population observed in the L valley.

In the hole kinetics, the initial rise of the main component of the hole transient (Fig. 5c) has been assigned to holes scattering from the split-off band into the heavy-hole band based on comparison of orbital characters in the VB (Fig. 1b) and only one intervalley scattering pathway that allows for a signal increase. This assignment is supported by comparison with hole relaxation times measured in germanium by optical pump-probe techniques. Woerner *et al*.^17^ reported intervalley scattering times of holes from the split-off band assisted by phonons to the heavy-hole band in the Γ valley to be within their instrument response time of 250 fs and estimated a lifetime of ∼100 fs by calculations. This value corresponds well to the (140±10) fs rise of the main component obtained in the present experiment. Although Woerner *et al*.^17^ excited specifically to the split-off band from the heavy-hole band near the Γ point, their ∼0.5 eV photon energy results in comparable hole energies as in the present experiment, considering that a broadband pulse with ∼1.65 eV central photon energy excites across the bandgap (≥0.8 eV). Due to the higher effective mass of the heavy holes, the scattering process towards the heavy-hole band is expected to be dominant and the model calculations and experimental results in their paper suggest the interband scattering from the split-off band is more effective than interband relaxation. The long time decay of the hole signal on the order of ∼1.1 ps indicates the recombination of the holes with the electrons, which within the error bars have the same decay constant for the observed time window and accessible signal-to-noise ratio.

In the present experiment, the electrons and holes exhibit a similar lifetime, that is, the decay constants are ∼1.1 ps for both. A question that arises is whether the electrons and holes recombine or if they relax to states that are invisible for the experiment, for example, states of *s* orbital character. The *in situ* measurement of the band shift suggests an interpretation of carrier recombination within the observed timescale, because the band shift over time (cf. Fig. 6) also follows a behaviour that indicates a single exponential decay of the free carriers in the CB as is observed in the kinetic analysis of the carrier dynamics in the CB, with agreement of the time constants. The longest lifetime occurs in the lowest bands, compared to a shorter lifetime at higher energies, supporting that the carriers have first thermalized and subsequently recombine. A possible recombination mechanism that would support the observed fast decay rate suggests the involvement of intermediate trap states and a Shockley–Read–Hall recombination mechanism^29^,^36^,^37^. This can be understood based on the nanocrystalline samples. The nanocrystalline thin films have a grain size of ∼11 nm. Further, it is known that randomly oriented non-passivated nanocrystals exhibit a large number of trap states at the interfaces of the nanocrystallites. These trap states can act as recombination centres^38^. The recombination times are expected to be significantly accelerated compared to single-crystalline samples, since assisted by phonon scattering the carriers can quickly scatter into these trap states and subsequently recombine. Given the high carrier density initially excited, Auger recombination is expected to play an additional role, while time constants in the order of 5 ps would be expected from an Auger recombination process^39^.

The observed timescales are in good agreement with visible transient reflectivity measurements on germanium nanorods, where carrier lifetimes of ∼6 ps were measured for nanorods having a diameter of 18 nm (ref. 40), considering that grown nanorods in general are expected to have a lower number of trap states compared to annealed nanocrystalline thin films. Further, the smaller domain size in the present experiment and three-dimensional versus two-dimensional trap state arrangement locations can explain the comparatively shorter measured lifetimes.

In summary, this paper presents the study of ultrafast carrier relaxation in germanium using NIR-XUV pump-probe spectroscopy with few-femtosecond time resolution. It is demonstrated that transient absorption in the XUV yields clear, spectrally resolved signatures for the energy distribution of electrons and holes simultaneously. This study on carrier dynamics of germanium using broadband ultrafast XUV transient absorption spectroscopy provides direct observation of the relaxation of both the electron and hole distributions in a semiconductor material after broadband (500–1,000 nm) excitation, which is unprecedented in conventional optical pump-probe studies. The transient absorption of XUV is further decomposed into contributions from electronic SB, band shifts and excited state broadening. To analyse the carrier dynamics in germanium, a Fourier transform-based technique is devised to retrieve the contribution from a single spin-orbit state at the germanium M_4,5_ edge in the transient absorption data. Comparison with first-principles calculations supports the assignment of electron and holes in the retrieved carrier dynamics transient signal. Due to the high temporal and energy resolution, broadband ultrafast XUV transient absorption spectroscopy is an ideal tool for disentangling the contributions of relaxation and recombination processes. Clear signatures of hot carrier relaxation for both electrons and holes in germanium occurring below 200 fs is observed, which has been so far inaccessible through narrowband, pure optical/NIR pump-probe techniques. In addition, the presented experiment suggests that the retrieved time-dependent band shift can be decomposed into a heat-induced and, predominantly, a carrier-induced redshift of the CB. The heat-induced redshift allows for *in situ* characterization of the sample temperature. The temporal behaviour of the carrier-induced redshift supports the observed fast ∼1.1 ps carrier recombination of electrons and holes observed in the carrier dynamics. Moreover, scattering and relaxation rates can be measured in the time domain and by the additional high-energy resolution quantitative data can be retrieved for different parts of the band structure. This technique will be an invaluable tool for research on more complex semiconductor materials such as strained layers, heteroatomic, ternary, quaternary systems, junctions, two-dimensional material compounds and quantum confined systems, which are becoming increasingly important for applications such as solar energy production and highly efficient computer processors.

## Methods

### Experimental details and data analysis

VIS-NIR pump pulses with 5 fs pulse duration are used to optically excite germanium thin films. The nanocrystalline germanium films, fabricated by electron beam deposition and subsequent annealing, are 100 nm thick and supported by a 30-nm-thick silicon nitride membrane. The crystallites have an average size of 11 nm, see Supplementary Note 7 for extended characterization. A spectrally continuous XUV pulse spanning the germanium M_4,5_ edge is generated by high harmonic generation in xenon assisted by polarization-assisted amplitude gating gating^41^. The XUV pulses are used to probe the transient population in the VB and CB in a transmission geometry and are subsequently resolved by a flat-field spectrometer. By changing the time delay *τ* between the pulses the transient changes are measured. A shutter periodically blocks the VIS-NIR pump beam to obtain a differential absorption signal. The data set shown in the main text Figs 2 and 3 (panels a–c and black line in Fig. 6) and analysed in Figs 4 and 5 consists of five averages and was scanned with 0.6 fs time steps around time zero and with 3.3 fs time steps out to 1.5 ps. The additional intensities shown in Fig. 6 consist of 50 averages each with 26 fs time steps on an interval of *τ*=[−60, +550] fs. To compensate for time delay drifts in parallel to the germanium sample the differential absorption at the argon 3*s*3*p*^6^6*p* autoionizing state is measured^42^. The instrumental response time has an upper bound estimated to ∼6 fs by comparing these gas transient absorption measurements. At the same time, the spectrometer resolution can be estimated to *δE*≈0.07 eV from known linewidths in gas absorption spectra. In the postexperimental data analysis, first the measured differential absorption data Δ*A*_meas_(*E*,*τ*) data are decomposed into contributions from SB, band shifts and excited state broadening. The high-quality static absorbance used for decomposing these contributions was measured by averaging one hundred transmission spectra alternating between the sample and an empty silicon nitride membrane under otherwise identical conditions to those for the time-resolved measurements. The SB signal is subsequently separated into a single spin-orbit split state by dividing out a constant phase factor in the Fourier domain originating from the spacing (Δ*E*_SO,Ge_=0.58 eV, ref. 23) and degeneracy of the core levels (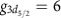 and 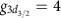) exhibiting spin-orbit splitting and subsequent back transformation:


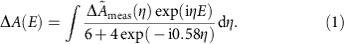


The retrieval constitutes the signal probed from the 3*d*_5/2_ state. Further details are given in Supplementary Note 4.

### Excited carrier density

The number of excited electrons has been estimated in two ways. First, from the fluence *F* of the laser pulse with central frequency *ν* and an absorption coefficient *α* of a sample with thickness *d*, one can estimate for a one-photon transition, where one absorbed photon excites one electron, that 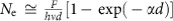. For the presented experiment (*I*=2 × 10^11^ W cm^−2^, *F*=12.4 × 10^−3^ J cm^−2^, *d*=100 nm, *α*=49 × 10^3^ cm^−1^, *λ*_0_=760 nm) one gets *N*_e_≅8 × 10^20^ cm^−3^. Second, the performed TDDFT calculations using **k**-dependent excitation and the spectrum of the experimental laser pulse an excitation fraction of 0.3% was calculated for a laser intensity of 2 × 10^11^ W cm^−2^. Using that a unit cell in germanium has a volume of *V*_0_=4.527 × 10^−23^ cm^3^ and that there are two atoms or eight valence electrons in this volume, one gets a valence electron density of *d*_0_=1.76 × 10^23^ cm^−3^. Hence, for the TDDFT-based calculation one gets *N*_e_≅5 × 10^20^ cm^−3^.

### Heat-induced band shift

In the time-dependent transient absorption experiments a transient signal at negative time delay, that is, NIR pump arrives after XUV probe, was consistently observed when measuring first the unexcited (cold) sample transmission followed by the excited (hot) sample spectrum to obtain a differential absorption spectrum. A comparison of the absorption change in a heated Ge film to this spectral feature suggests that it is related to an increased temperature of the germanium thin film after optical excitation causing a heat-induced band shift^43^. The absorption edge redshifts causing a positive differential signal. By resolving the underlying shift due to thermal expansion of the lattice Δ*E*_ren,therm_, a temperature can be assigned to the thin film. For example, for an average pump power of Δ*P*_avg_=0.39 mW the measured shift of the edge is Δ*E*_ren,therm_=72.8 meV, suggesting a temperature of *T*=475 K, which is confirmed by heat diffusion calculations using a finite element method (Supplementary Note 5).

### First-principles TDDFT and XAS calculations for germanium

To support the assignment of spectroscopic features, X-ray absorption spectroscopy calculations based on the eXcited-electron Core-Hole (XCH)^44^ approach were combined with first-principles TDDFT^45^,^46^. This first-principles treatment of the electric field dynamics described using real-time TDDFT^45^,^46^ includes effects beyond the linear response of the material, and similar theoretical treatments reproduce electron tunnelling in silicon^10^ and the dielectric breakdown in insulators^47^. The exciting VIS-NIR pump laser field models a few-femtosecond, 780 nm pulse with a peak intensity of 1 × 10^11^ W cm^−2^ (see Supplementary Fig. 7a).

The excited-state electron and hole occupations generated by interaction with the pump laser pulse are obtained from the real-time TDDFT calculation in the form of a single-particle density matrix. This one-electron density matrix is subsequently projected onto an eigenbasis of the core-excited XCH Hamiltonian, along the lines described previously^10^,^48^,^49^, to estimate the M_4,5_ edge XUV absorption in the presence of excited carriers. The difference between this excited state spectrum and the corresponding ground state spectrum in the absence of electron–hole pairs is compared to the 3*d*_5/2_ core-level differential absorption retrieved from experiment at time delays directly after excitation, shown in Fig. 3c. Inclusion of the core–hole excitonic effects, at the level of the XCH approach, in the core-level absorption calculation was necessary to match the magnitude of the negative feature at 30 eV. In the first-principles calculations, the excited and ground state spectra are treated only for a single spin-orbit split state and shifts, such as core-level shifts or band shifts, are not included. We note that at the level of the adiabatic TDDFT and XCH theories employed here we expect state-blocking effects in the excited state M_4,5_ edge absorption to be captured but many-body effects such as excited-state bandgap shifts and carrier lifetime modulation are not described. Therefore, the comparison with experiment is invoked primarily in the context of state blocking where good agreement is observed (Fig. 3c). Further details are given in Supplementary Note 10.

### Singular value decomposition

The carrier dynamics can be decomposed into several carrier distributions with independent relaxation dynamics. Under such formulation, the transient absorption signal Δ*A*(*E*,*t*), with *E* as photon energy and *t* the delay time, can be decomposed into


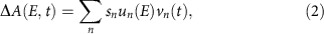


where *u*_*n*_ is the *n*th component of transient absorption, which corresponds to a distinct carrier distribution and *ν*_*n*_ its accompanying relaxation dynamics. *s*_*n*_ is the singular value of the *n*th component, which signifies the importance of the component on the overall dynamics.

### Germanium band structure calculation

The band structure of germanium (Fig. 1b) is calculated by DFT within the Vienna *ab initio* simulation package^50^,^51^, using a generalized gradient approximation and Hubbard (GGA+U) approach. The computational method and parameters are detailed in ref. 52.

### Data availability

The experimental data can be obtained on reasonable request from the corresponding authors.

## Additional information

**How to cite this article:** Zürch, M. *et al*. Direct and simultaneous observation of ultrafast electron and hole dynamics in germanium. *Nat. Commun.* **8,** 15734 doi: 10.1038/ncomms15734 (2017).

**Publisher's note:** Springer Nature remains neutral with regard to jurisdictional claims in published maps and institutional affiliations.

## Supplementary Material

Supplementary Information Supplementary Figures, Supplementary Notes and Supplementary References
