# Supplementary material for: Direct and simultaneous observation of ultrafast electron and hole dynamics in germanium
Source: Nat Commun. 2017 Jun 1;8:15734. doi: 10.1038/ncomms15734 (PMC5461502; doi:10.1038/ncomms15734)
Supplement: Supplementary Information — Supplementary Figures, Supplementary Notes and Supplementary References [file ncomms15734-s1.pdf]

Supplementary Note 1: Extended Experimental Methods

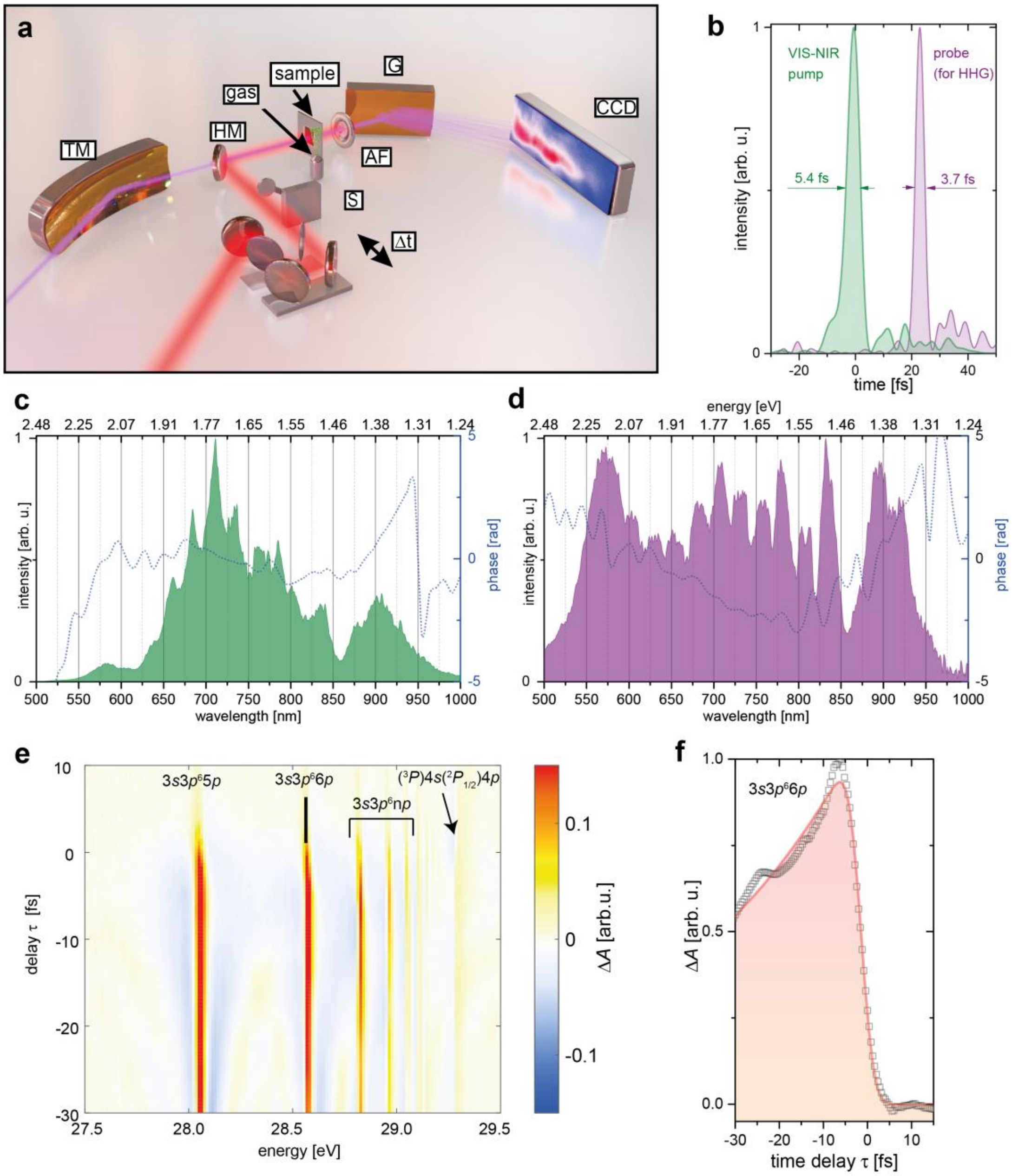

Supplementary Figure 1: *Detailed experimental setup and pulse characterization. (a) The extreme ultraviolet (XUV) probe pulse is refocused by a toroidal mirror (TM) onto the sample. A grating (G) disperses the XUV which is measured with a charge-coupled device (CCD). The visible-to-near infrared (VIS-NIR) pump pulse is time delayed with respect to the XUV pulse and focused onto the sample. Both*

beams are collinearly recombined by means of a hole mirror (HM). An aluminium filter (AF) blocks the VIS-NIR light before the spectrometer. A shutter (S) in the pump arm periodically blocks the pump light for differential absorption measurements. (b) Time domain characterization of the VIS-NIR pump and probe pulse (used for high harmonic generation) in violet and green colour, respectively. Panels (c) and (d) show the corresponding spectra and spectral phase. The pump pulse has a smaller bandwidth and hence a slightly longer pulse duration due to limitations on the broadband coating on the beamsplitter used to separate pump and probe arm. (e) Exemplary measured transient absorption (TA) signal of the 3s3p^{6}np 1P autoionization series of argon. (f) The 3s3p^{6}6p transient signal (spectral position marked by black line in (e)) is employed for time drift compensation and can be used for a time resolution estimate by fitting a Gaussian convoluted with an exponential decay (solid line and shaded area), see text for details.

In Supplementary Fig. 1a the XUV transient absorption apparatus is illustrated. Carrier envelope phase (CEP) stabilized pulses centred at 790 nm, 25 fs in duration are produced using a commercial Ti:sapphire chirped pulse amplifier at a repetition rate of 1 kHz. After spectrally broadening these pulses in a neon-filled hollow-core fibre, these pulses are further compressed via chirped mirrors (PC70, Ultrafast Innovations) to approximately 4 fs pulse duration. A chopper before the beam is split by a 90/10 beam splitter into the probe and pump arm, respectively, reduces the repetition rate to 100 Hz to reduce the average power, minimizing damage of the solid-state sample from heating by successive pulses. Fused silica (FS) wedges in both the pump and probe arm allow for fine-tuning the dispersion. The proper compression of the pulses is confirmed by the dispersion scan technique[1].

The probe arm proceeds through a 75 μm thick quartz plate followed by a quarter-wave plate for polarization-assisted amplitude gating (PASSAGE) gating[2] and is subsequently focused inside a 1 mm gas cell filled with 28 torr of xenon gas to generate high harmonics. The residual VIS-NIR in the probe arm is blocked by a 200 nm thick aluminium filter. A gold-coated toroidal mirror focuses the XUV by direct imaging the source onto either a solid-state thin film or another 1 mm gas cell for characterization

measurements. The focal spot size of the XUV radiation is estimated to have a diameter of approximately 50 μm full-width half maximum (FWHM) by a knife-edge scan. The XUV light that is transmitted through the sample is measured by a flat-field spectrometer. The spectrometer was calibrated using argon and neon autoionizing states between 26-48 eV (refs. 3,4), and the spectral resolution was determined to be approximately 70 meV. The typical XUV continuum spans 25 to 40 eV (see main text Fig. 1d) and exhibits CEP effects if the CE is scanned, indicating the attosecond or very short attosecond pulse train nature of the pulse.

The VIS-NIR pump pulse propagates alongside the vacuum apparatus and is collinearly recombined with the XUV beam by means of an annular mirror after the toroidal mirror. The VIS-NIR pump pulse covers photon energies from 1.2 to 2.5 eV (Supplementary Fig. 1b) and can be blocked by a shutter in order to measure the absorption change with and without the pump pulse. The VIS-NIR pump arm is focused onto the target with a beam diameter of approximately 100 μm (FWHM). A piezo-driven delay stage in the pump arm allows for precise control of the relative time delay between the pump and probe pulses. An additional 200 nm thick aluminium filter after the sample blocks the pump arm before the spectrometer. An iris is used for regulating the pump arm to specified intensities.

To correct for thermally induced time delay drifts, the experiment alternates between measuring pump off and pump on transmission spectra on the germanium sample and an argon gas cell for each individual time step. The germanium sample was raster scanned to randomly chosen positions on a regular grid of points spaced by 150 microns in both dimensions over an area of approximately 2.5 x 2.5 mm² to ensure averaging out possible local inhomogeneities of the thin film. Typical integration times are on the order of one second to optimally use the dynamic range of the detector. However, for signal levels in the $\Delta A < 0.05$ level, averaging several data sets is required for improving the signal-to-noise ratio. The typical number of averages range from 5 to 50, which takes between 4 and 24 hours for a complete experiment at all time delays, depending on the number of time steps. Hence, time delay drifts in the experimental apparatus must be corrected for. Here, it was chosen to scan all time delays by alternating

between the germanium sample and the argon gas cell and repeat the full scan for a number of averages. The $3s3p^6 6p$ autoionizing state in argon produces a sufficiently strong transient signal[5] in each single scan (Supplementary Fig. 1e and f). Fitting a Boltzmann sigmoidal function to the normalized rise of this feature

$$\Delta A_{\mathrm{Ar},3s3p^6 6p}(t) = \frac{1}{1+\exp[(t-t_0)/dt]}, \tag{1}$$

where t_0 and dt are the time of the signal reaching the 50% level and the slope at that point, respectively, allows determining a common time base. Although this approach does not reflect the complexity of field-induced line shape changes,[6] nor does it produce an absolute time zero[7], it allows an effective determination of the time delay drifts between different scans in situ on target and without changing the experimental scheme. Further, monitoring dt allows to confirm constant pump pulse characteristics over the course of the experiment, assuming that slight differences in pump intensity would modify the ponderomotively driven line shape changes significantly. For the experiments presented here, typical time delay drifts between successive scans were on the order of one femtosecond, which were compensated in post-experimental analysis by interpolating all data sets onto a common time delay axis. For estimating the temporal resolution of the setup, a Gaussian convoluted with an exponential decay truncated at time zero is fit to the transient at the $3s3p^6 6p$ line centre[6] (Supplementary Fig. 1f). The resulting width of the Gaussian at full-width-at-half-maximum (FWHM) of $\tau_{\mathrm{XUV}\otimes\mathrm{VIS\text{-}NIR}} = 5.4 \pm 0.2$ fs corresponds to the cross-correlation between the VIS-NIR pulse and the broadband XUV pulse. Given that the VIS-NIR pulse was characterized to $\tau_{\mathrm{VIS\text{-}NIR}} = 5.4 \pm 0.5$ fs by the D-scan technique (Supplementary Fig. 1b), it can be concluded that the VIS-NIR pulse is properly compressed on target and that the broadband XUV pulse at worst consists of a very short attosecond pulse train with a FWHM of $\tau_{\mathrm{XUV,worst\ case}} = \sqrt{\tau_{\mathrm{XUV}\otimes\mathrm{VIS\text{-}NIR}}\big|_{\max}{}^2 - \tau_{\mathrm{VIS\text{-}NIR}}\big|_{\min}{}^2} \approx 2.7$ fs resulting in the upper bound for the instrumental response function of approximately 6 fs mentioned in the main text.

Supplementary Note 2: Calculation of k-dependent excitation cross section

The measured change of absorbance ΔA_{meas} in the XUV is proportional to the population of holes, N_{h}, and electrons, N_{e}, in the valence and conduction bands. The population of holes is proportional to the oscillator strength $\Omega(\mathbf{k})$ and an excitation probability P, assuming a uniform oscillator strength of unity and therefore determined by the spectrum of the VIS-NIR pump pulse $I(\omega)$ and the energy gap between valence and conduction bands, $\omega_{\mathrm{g}}(\mathbf{k})$:

$$N_{\mathrm{h}}(\mathbf{k}) \sim \Omega(\mathbf{k}) P, \tag{2}$$

and

$$P(\mathbf{k}) = I(\omega) \sum_{\{i,j\}} \delta(\omega - \omega_{\mathrm{g}}^{ij}(\mathbf{k})), \tag{3}$$

where i and j are band indices for valence and conduction bands, respectively. Supplementary Figure 2 shows the excitation probability at different $\mathbf{k}$ points in the heavy-hole, light-hole, and split-off bands. The calculation includes excitation to the four low-lying conduction bands and assumes a constant transition dipole element.

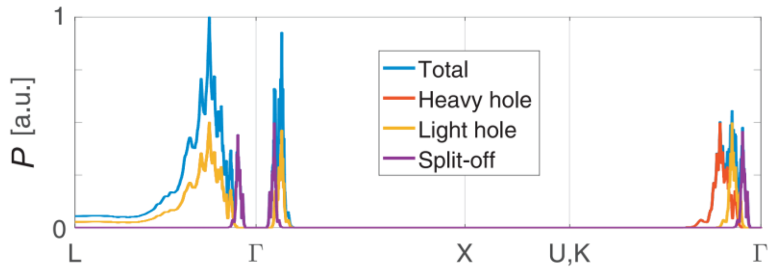

Supplementary Figure 2: Allowed hole excitations at different **k** *points in the heavy-hole and light-hole and split-off band and the total contribution from the three for the visible-to-near infrared (VIS-NIR) pulse used in the experiment for germanium. Note that between the L and X points the heavy and light hole band are degenerate, i.e. the yellow line covers the red line.*

Supplementary Note 3: Extraction of state blocking, band shifts and broadening in time-dependent differential transient absorption data at the Ge $M_{4,5}$-edge

The assignment of features in a measured raw ΔA_{meas} trace is hampered by several physical effects that overlap spectrally. The change of absorbance (ΔA_{meas}) includes contributions from electronic state blocking from carrier excitation, spectral shift of the M-edge due to core-level shifts and band shifts from excited electron-hole plasma and phonons, and spectral broadening of the excited state spectrum. To retrieve the carrier dynamics from the transient absorption signal, we decompose the measured change of absorbance, $\Delta A_{\mathrm{meas}}(E,\tau)$, (see main text Fig. 2a) into contributions from electronic state blocking ΔA_{SB}, spectral shift $\Delta A_{\mathrm{shift}}(E, \Delta E_{\mathrm{shift}}(\tau))$ of the static absorption spectrum ($A(E)$, main text Fig. 2b) by energy $\Delta E_{\mathrm{shift}}$, and spectral broadening $\Delta A_{\mathrm{broad}}(E, \sigma(\tau))$. The broadening is modeled by a Gaussian broadening of the static absorption spectrum by width σ. Regarding the broadening, assuming a Gaussian functional for broadening relates to phonon-induced broadening while a Lorentzian functional relates to electronic-induced broadening. Since for the presented experiment the component due to broadening in general was found to be negligible, the functional due to broadening was not further evaluated, although it was retained in the analysis because for other materials or parameters it could become important. We fit the measured change of absorption at each time delay with weighted numerical minimization of the following function over the energy range $E = [27.5, 30.5]$eV:

$$\epsilon_n = \left|\Delta A_{\mathrm{meas}}(E,\tau) - \Delta A_{\mathrm{broad,n}}(E,\sigma(\tau)) - \Delta A_{\mathrm{shift,n}}(E, \Delta E_{\mathrm{shift}}(\tau)) - \Delta A_{\mathrm{SB,n\text{-}1}}(E,\tau)\right|, \qquad (4)$$

where n denotes the step in the iterative minimization procedure, and

$$\Delta A_{\mathrm{broad,n}}(E,\sigma(\tau)) = A(E) \star \exp(-E^2/[2\sigma(\tau)^2]) - A(E) \qquad (5)$$

$$\Delta A_{\mathrm{shift,n}}(E, \Delta E_{\mathrm{shift}}(\tau)) = A(E - \Delta E_{\mathrm{shift}}(\tau)) - A(E), \qquad (6)$$

where the star operator denotes a convolution. The initial guess for the transient absorption induced by state blocking $\Delta A_{\mathrm{SB,0}}(E,\tau)$ is modelled as follows.

As a first guess to seed the iterative algorithm, we assume the initial carrier distribution resembles Gaussians and their spin-orbit contributions to the transient absorption signal can be written as

$$\begin{aligned}\Delta A_{\mathrm{SB},0}(E,\tau) = \frac{|\Delta A_{\mathrm{meas}}(28.3\text{ eV},\tau)|}{\max|\Delta A_{\mathrm{meas}}(28.3\text{ eV},\tau)|}\Bigg(& 6\ \exp\left\{-\left[\left(E-E_{\mathrm{F}}-\frac{E_{\mathrm{c}}}{2}\right)/\Delta E_{\mathrm{BW}}\right]^2\right\} \\ &+ 4\ \exp\left\{-\left[\left(E-E_{\mathrm{F}}-\frac{E_c}{2}-\Delta E_{\mathrm{SO}}\right)/\Delta E_{\mathrm{BW}}\right]^2\right\} \\ &+ 6\ \exp\left\{-\left[\left(E-E_{\mathrm{F}}+\frac{E_c}{2}\right)/\Delta E_{\mathrm{BW}}\right]^2\right\} \\ &+ 4\ \exp\left\{-\left[\left(E-E_{\mathrm{F}}+\frac{E_c}{2}-\Delta E_{\mathrm{SO}}\right)/\Delta E_{\mathrm{BW}}\right]^2\right\}\Bigg).\end{aligned} \tag{7}$$

Electrons and holes contribute two Gaussians each in the above expression due to spin-orbit splitting ΔE_{SO} in the 3*d* core level. The pre-factors 6 and 4 originate from the degeneracies of the 3*d* core levels. It is important to mention here, that this step of generating a seed for the state blocking contribution in the iterative algorithm and taking spin-orbit splitting into account is essential, since we choose to decompose the components by using the measured raw transient absorption signal and the static absorbance, both of which have contributions by spin-orbit splitting in germanium. In Sec. S4 of this document we indicate how in a step subsequent to retrieving the state blocking component by the iterative procedure outlined here the spin-orbit contributions are separated. Assuming a Gaussian shape of the state blocking here, as first approximation, is chosen as a reasonable compromise between the energy resolution of the instrument and the initial distribution expected from the spectrum of the excitation pulse, which would translate into a Fermi-Dirac type distribution after thermalization. Here, we assign the Fermi energy $E_{\mathrm{F}} = 29.2$ eV, the average optical excitation energy $E_{\mathrm{c}} = 1.65$ eV, and a bandwidth of excitation $\Delta E_{\mathrm{BW}} = 1$ eV according to the experimental parameters. The state-blocking-contributed transient absorption signal is refined and retrieved at every iteration step n, using

$$\Delta A_{\mathrm{SB,n}}(E,\tau) = \Big[2\left(\Delta A_{\mathrm{SB,n\text{-}1}}(E,\tau)\right) + \left(\Delta A_{\mathrm{meas}}(E,\tau) - \Delta A_{\mathrm{broad,n}}(E,\sigma(\tau)) - \Delta A_{\mathrm{shift,n}}(E,\Delta E_{\mathrm{shift}}(\tau))\right)\Big]/3 \tag{8}$$

where the weighting factors are chosen to aid smooth convergence. Note that during the iterations no particular treatment of spin-orbit splitting is necessary, since all components considered and input into the procedure have contributions by the spin-orbit split 3*d* core level in germanium. In this way the iterative procedure works as closely as possible on the experimentally measured data, i.e. the transient absorption signal and the static absorbance, which seeks to improve numerical stability of the procedure.

After five iterations a stable state blocking signal is retrieved (main text Fig. 2c) along with a delay dependent broadening $\sigma(\tau)$ and $\Delta E_{\mathrm{shift}}(\tau)$. In main text Fig. 2d and e the resulting contributions for $\Delta A_{\mathrm{broad}}(E,\sigma(\tau))$ and $\Delta A_{\mathrm{shift}}(E,\Delta E_{\mathrm{shift}}(\tau))$ are shown. Supplementary Fig. 3 depicts the first iteration of the decomposition. Already with the first guess for the state blocking based on measured experimental parameters, reasonable agreement between the measured $\Delta A_{\mathrm{meas}}(E,\tau)$ trace and a calculated trace based on $\Delta E_{\mathrm{shift}}(\tau)$ and $\sigma(\tau)$ after the first iteration is achieved (Supplementary Fig. 3a & b). The major contribution besides state blocking comes from an underlying redshift (Supplementary Fig. 3c). The decomposition suggests that broadening appears to play a minor role and contributes less than 5% to the signal. From the retrieved state blocking, i.e. when the iterative procedure has converged, one can subsequently retrieve the spin-orbit separated state blocking by applying the method outlined in Section S4 (main text Fig. 2f); the result can then be further analysed for the underlying carrier dynamics. It is important to note that this model only assumes a linear shift for all energies. As can be also seen for the analysis of the heat-induced band shift (Supplementary Fig. 5b), the agreement of the experiment and model is reasonable below approximately 31 eV. At higher energies the disagreement can be explained by different critical points in the band structure, which shift by different amounts compared to the main contributions at the bottom of the CB. Hence, a more sophisticated model would be required to model the behaviour of these critical points lying higher in energy. Further, for a band gap renormalization, one

would expect a symmetric shift around the Fermi energy, i.e. the conduction band shifting down and the valence band shifting up by the same amount. In the presented model this means one would need to introduce more fitting parameters to account for the different sign of shifts for different energies. Also the contributions of spin-orbit splitting would need to be taken care of, i.e. complex shifts in the region where the CB of one spin-orbit state overlaps with the VB of the other. Since the VB state blocking signal emerges in a relatively flat region of the static absorbance between 28 and 29 eV, these shifts of the underlying absorbance with the wrong sign only lead to small errors with less than 5% magnitude (see slight blueish colour code between 28 and 29 eV in main text Fig. 2e). This is also the reason as to why in the raw measured transient absorption trace (main text Fig. 2a) the VB state blocking signal can be appreciated as a separate feature.

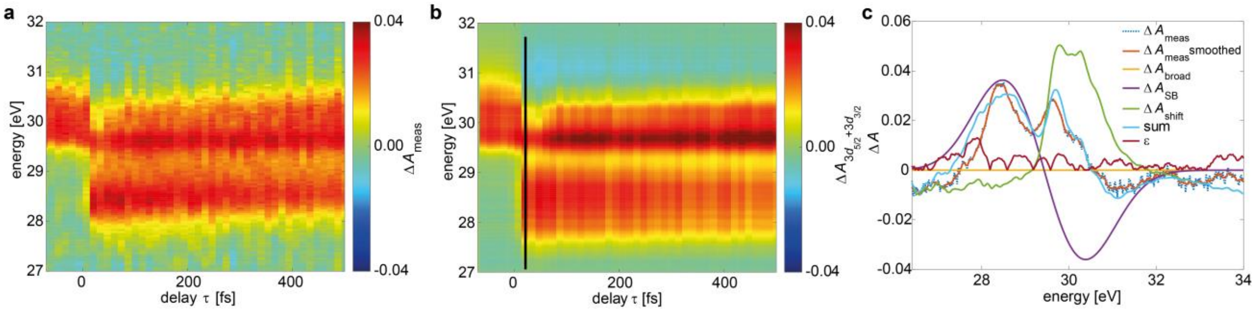

<u>Supplementary Figure 3:</u> *Comparison of measured differential absorption trace with first iteration of the decomposition model. During the first iteration two Gaussian distributions with magnitudes comparing to the 28.3 eV measured signal are assumed and* $\Delta E_{\mathrm{shift}}(\tau)$ *and* $\sigma(\tau)$ *are retrieved for each time step. Comparing the measured differential absorption trace (a) with a forward calculated trace (b), i.e.* $\Delta A_{\mathrm{SB,0}}(E,\tau) + \Delta A_{\mathrm{broad,n}}(E,\sigma(\tau)) + \Delta A_{\mathrm{shift,n}}(E,\Delta E_{\mathrm{shift}}(\tau))$, *already shows reasonable agreement. Investigating the components for a single time step in panel (c) shortly after time zero (black line in b) shows that the first guess of Gaussian distributions for the state blocking* (ΔA_{SB}) *and an absorption change induced by the red-shift of the spectrum by 110 meV* ($\Delta A_{\mathrm{shift}}$)*, which comprises heat-induced and photon-induced band shifts and possibly carrier-induced core level shifts, are the main contributions that minimize the error* (ϵ) *between the measured* (ΔA_{meas}) *and forward calculated signal* (sum)*.*

Supplementary Note 4: Separation of spin-orbit components

The measured transient absorption of Ge at the $M_{4,5}$-edge as a function of energy, $\Delta A_{\mathrm{meas}}(E)$, is contributed by the transitions from the $3d_{3/2}$ and $3d_{5/2}$ levels, split by 0.58 eV (ref. 8), to the valence and conduction band. Because the core-level states are localized with fixed energy, one can approximate ΔA_{meas} as

$$\Delta A_{\mathrm{meas}}(E) = 6\Delta A(E) + 4\Delta A(E - 0.58), \tag{9}$$

where $\Delta A(E)$ is the contribution of a single spin-orbit split core-level state while 6 and 4 are the degeneracy of the $3d_{3/2}$ and $3d_{5/2}$ levels, respectively. It is assumed that the transition strengths follow those degeneracies. Transforming $\Delta A_{\mathrm{meas}}(E)$ into its Fourier counterpart $\Delta\tilde{A}_{\mathrm{meas}}(\eta) = \int e^{-\mathrm{i}\eta E}\Delta A_{\mathrm{meas}}(E)\mathrm{d}E$, the previous equation becomes

$$\Delta\tilde{A}_{\mathrm{meas}}(\eta) = \Delta\tilde{A}(\eta)\times\left(6 + 4\exp(-\mathrm{i}0.58\eta)\right). \tag{10}$$

The shifted and factorized contribution of the one spin-orbit split state relative to the other manifests itself as a constant and known phase factor, which can be divided out. By inverse Fourier transform, the contribution of the signal from a single spin-orbit core-level state is retrieved as

$$\Delta A(E) = \int \frac{\Delta\tilde{A}_{\mathrm{meas}}(\eta)\exp(\mathrm{i}\eta E)}{6 + 4\exp(-\mathrm{i}0.58\eta)}\mathrm{d}\eta. \tag{11}$$

The scheme of spin-orbit decoupling is illustrated in Supplementary Fig. 4. Supplementary Figure 4a shows the measured data $\Delta A_{\mathrm{meas}}(E)$ versus pump-probe delay, where the features from the two spin-orbit contributions overlap together. Supplementary Fig. 4b illustrates how the same but factorized signal from two shifted states add up causing a broader feature. Supplementary Figure 4c is the spin-orbit separated data containing only the excitation from the $3d_{5/2}$ level. The features clear up and more details become visible.

The separation method described here is sensitive to the degeneracy of the spin-orbit states and the spin-orbit energy splitting. Due to the involvement of an imaginary component in the Fourier transform, artefacts will arise as non-zero imaginary parts in $\Delta A(E)$. Hence, the zero value of the imaginary part in $\Delta A(E)$ signifies the success in decoupling the contribution of a single spin-orbit state with this method. In the presented experiment it was useful to retrieve the state blocking contribution to the measured differential absorption signal first (cf. Section S3) and apply the spin-orbit separation only to the state blocking contribution to extract time dynamics of the holes and electrons.

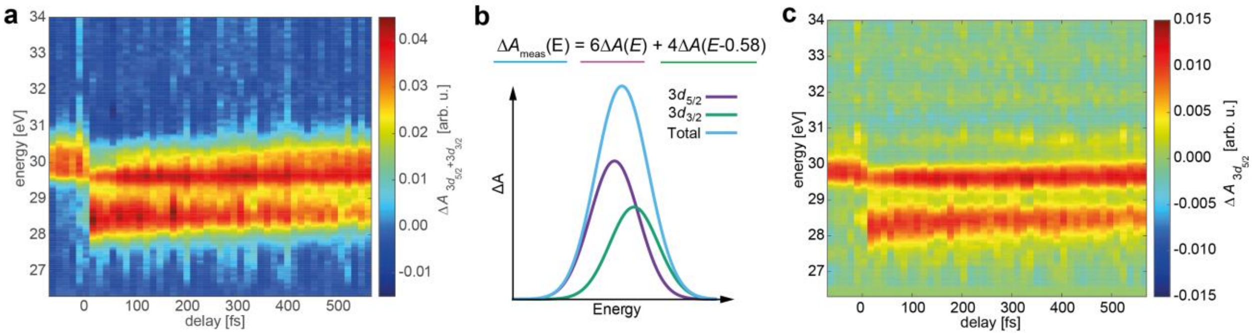

<u>Supplementary Figure 4:</u> *Demonstration of decoupling of spin-orbit components. (a) The* $3d_{5/2}$ *and* $3d_{3/2}$ *states in germanium cause smeared out transient absorption traces since the spin-orbit splitting of* $\Delta E_{so}=0.58$ *eV nearly matches the band gap. (b) The measured signal* $\Delta A_{meas}(E)$ *is the result of the addition of copies of the same signal* ΔA *shifted by the spin-orbit splitting and factorized by the degeneracy of the states (6 and 4 for the* $3d_{5/2}$ *and* $3d_{3/2}$ *states in germanium, respectively). (c) Using the Fourier method described in the text, one can retrieve a transient absorption trace separated for a single spin-orbit split state.*

<u>Supplementary Note 5: Identification of a heat-induced feature in transient absorption data</u>

At a repetition rate of 100 Hz, the germanium sample has 10 ms to relax between consecutive laser pulses, which is considered sufficient for electronic relaxation. Still a transient feature is observed for negative time delays (see Supplementary Fig. 5a), which can be explained by heat accumulated in the sample during exposure. The 100 nm thick germanium film on a 30 nm thick silicon nitride membrane is thermodynamically nearly a two-dimensional object, and heat can only dissipate in the plane to the thicker silicon wafer that frames the membrane. Measuring the negative time delay feature, which we refer to as heat-induced feature, depending on the pump energy, or average power (Supplementary Fig. 5a), reveals a near-linear increase of the signal with average power. The origin of the heat-induced feature can be understood by noting that differential absorption is measured, which inherently is sensitive not only to absolute signal changes but also to energy shifts of the absorbance. Supplementary Fig. 5b shows a differential absorption signal (solid line) calculated from the static absorbance measured on the germanium thin films (see main text Fig. 1d) and a copy of the same signal shifted by 72.8 meV in comparison to the measured heat-induced feature (dotted line) at 0.39 mW average power in the pump

arm. The agreement of amplitude and shape confirms that the feature to first order originates from a heat-induced band shift induced by the increased temperature. Using an empirical formula for temperature induced redshift of the conduction band[9]

$$\Delta E_{\text{indirect gap}} = a - \frac{\alpha T^2}{\beta + T} \tag{12}$$

with the parameters $a = 0.741\text{eV}$ s, $\alpha = 4.561\times10^{-4}\text{eVK}^{-1}$ and $\beta = 210\text{K}$ for the lowest lying critical point in the conduction band of germanium allows an estimate the temperature of the thin film for different average pump powers by extracting the induced energy shift from the measured heat-induced feature (Supplementary Fig. 5c). Note that we chose to neglect core-level shifts here, which is reasonable because in contrast to carrier-induced band shifts, where small core-level shifts are expected (cf. Sec. S3), the heat-induced band shifts are expected to have only little impact on the core-level screening. It can be seen that the residual temperature of the thin film is significantly elevated over room temperature even at fractions of a milliwatt average power. From experimental observation, laser annealing sets in at temperatures over 500 K. At pump energies larger than 6 µJ, i.e. 0.6 mW average power, permanent damage of the thin films was observed either by complete destruction of the thin film or by changed static absorbance, indicating a change of structure. To confirm the observation further, a simulation of the heat dissipation using COMSOL was performed. A continuous-wave source of the same average power and 100 µm diameter (FWHM) was assumed for a model of the germanium-silicon-nitride layer system. The three dimensional heat diffusion and radiation losses were considered. Plotted in the inset in Supplementary Fig. 5c are the retrieved peak temperatures at the excitation spot in comparison to the measured temperatures. The heat diffusion is modelled by a finite element method (COMSOL) with heat conductivity of Ge taken as 5.7 $\text{Wm}^{-1}\ \text{K}^{-1}$ (ref. 10). The slight deviation in slope can be explained by differing heat conductivities from the literature value[10] for nano to polycrystalline germanium and the grain size in the particular samples. The COMSOL simulation further revealed that the temperature in the hot spot builds up within the first few laser pulses. Hence, it is a good assumption that the data presented

in this paper were collected at a given temperature and the temperature gradient during the measurement was marginal.

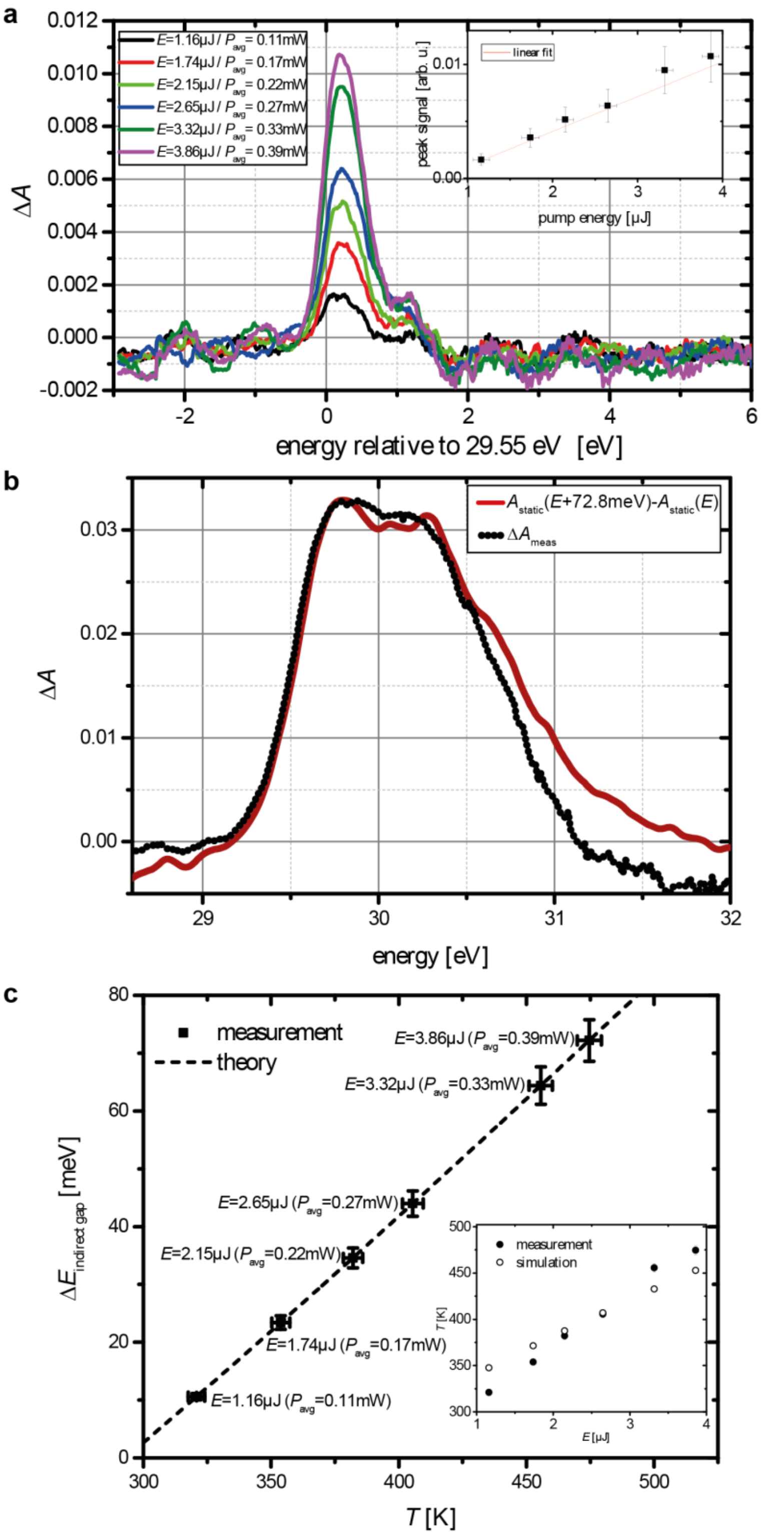

<u>Supplementary Figure 5</u>: *Negative time delay feature manifesting as temperature induced shift. (a) For increasing pump energy or average power the heat feature increases nearly linearly. (b) The origin can*

be explained by a heat-induced band shift, which is confirmed by comparing the measured heat-induced feature with a differential absorption calculated from the measured absorption of the thin film and a shifted copy. In this example for 0.39 mW average power best agreement is achieved for a shift of 72.8 meV. (c) From the measured shift the temperature of the thin film can be derived by using an empirical formula for the redshift. The result is compared to a heat dissipation simulation using COMSOL (inset in c).

The deviations on the high energy side in Supplementary Fig. 5b can be explained by different temperature coefficients for the higher energy critical points in the conduction band[9]. Hence, for full replication of the heat feature not only a linear shift but also higher order shifts causing modification of the absorbance at elevated temperature must be considered. The linear shift used here can be understood as a first order term for describing the band shift, which shall be sufficient for retrieving the temperature of the thin film and explaining the origin of this feature.

It is important to note that in the experiments always pump off, i.e. cold membrane, was measured first, followed by the pump on measurement, i.e. hot membrane. At each time delay point 100 laser pulses are accumulated for the pump off and pump on conditions, followed by moving a gas cell into the laser beam and the germanium sample out. The gas reference measurements were performed with the same number of laser pulses. Raster scanning was performed on a regular grid of illumination points spaced by 150 microns in both directions. These illumination points were randomly addressed, while the time delay was homogenously increased from negative to positive time delays with the step sizes indicated in the Methods section of the main text. These steps are performed to assure that the temperature of the thin film is relaxed at each new time delay.

Supplementary Note 6: Calculation of the induced band gap renormalization

For a given concentration of free carriers N_e the carrier-induced band gap renormalization, i.e. the combined amount of blueshift of the VB and redshift of the CB, can be calculated by[11]

$$\Delta E_{\mathrm{gap}} = -\left(\frac{e}{2\pi\varepsilon_0\varepsilon_{\mathrm{s}}}\right)\left(\frac{3}{\pi}\right)^{\frac{1}{3}} N_{\mathrm{e}}^{\frac{1}{3}}, \tag{13}$$

where ε_0 is the permittivity of free space and ε_s is the relative static dielectric constant of the semiconductor. In this work $\varepsilon_{\mathrm{s,Ge}} = 15.8$ is used[12]. For N_e values, the were derived by the TDDFT calculation including **k** space response to the used VIS-NIR pulses. The carrier-induced band gap renormalization scales with the third root of the number density of free carriers, due to the average interparticle spacing in three dimensions. In this work only the redshift of the CB is predominantly observed and retrieved by the model outlined in Sec. S3. Thus in the main text the time-dependent redshift of the CB is compared to $\Delta E_{\mathrm{gap}}/2$, which we refer to as band shift rather than a renormalization.

Supplementary Note 7: Sample preparation and characterization

The germanium sample is prepared by electron beam physical vapour deposition (Lebow Company). One hundred nanometre germanium thin films are deposited onto 30 nm thick silicon nitride windows. The germanium film is subsequently annealed at 450 °C for 3 hours. X-ray diffraction (XRD) patterns were acquired using a Bruker GADDS Hi-Start D8 diffractometer with a Co anode at 45 kV/35 mA (λ=1.79 A). The peak positions and peak widths of the pattern were determined using a commercial software package (Philips X'Pert HighScore Plus). The XRD pattern of Ge was simulated using the peaks (111, 022, 113, and 222) and those were well matched with reference pattern of cubic Ge (ICSD# 98-060-0744). The average grain size (approximately 11 nm) was estimated from X-ray peak broadening using the Scherrer equation after correction for instrumental broadening.

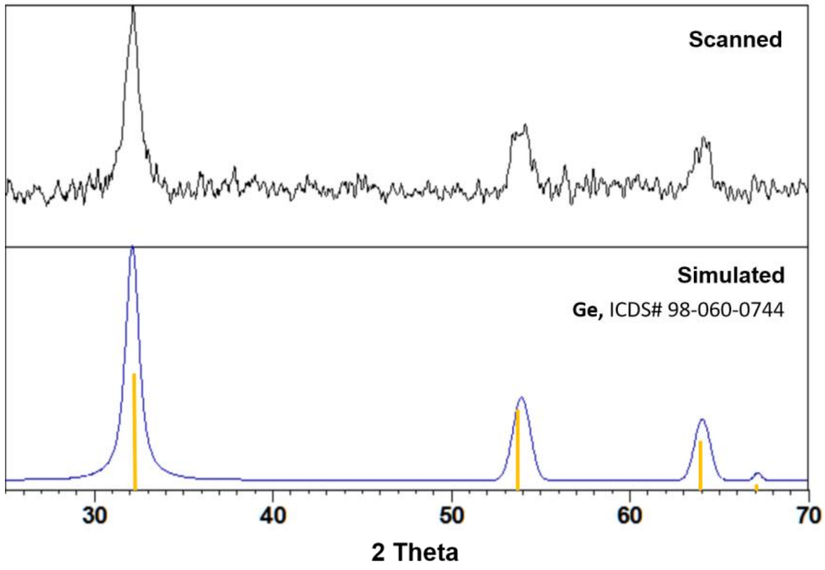

Supplementary Figure 6: X-ray diffraction (XRD) pattern collected from the Ge film grown on Si_3N_4 window (upper) and simulated pattern from the peaks fitted to the XRD pattern (lower).

Supplementary Note 8: Intensity calibration for the pump pulse

To obtain the peak intensity of the pump pulse, we measured the pulse energy E_p at the focus. The pump beam profile features a central spot and a ring originating from diffraction at the hole of the annular mirror that is used for collinear recombination with the XUV. Assuming a Gaussian beam profile at the central spot, the peak intensity of the excitation pulse in vacuum can be written as

$$I_\mathrm{vac} = \frac{4\sqrt{\ln 2}}{\pi\sqrt{\pi}} \frac{f_\mathrm{b} f_\mathrm{t} E_\mathrm{p}}{w_\mathrm{x} w_\mathrm{y} \tau_\mathrm{p}}, \tag{14}$$

where w are the half beam diameter for the long and short axes of the elliptical central spot, τ_p is the excitation pulse duration, f_b is the fraction of pulse energy in the central spot, and f_t is the fraction of energy of the main pulse in the temporal domain.

The temporal profile of the pump pulse is characterized by dispersion scan (D-Scan, Sphere Photonics)[1]. The pulse characterization reveals the pump pulse duration at approximately 5 fs and energy fraction in the main pulse $f_\mathrm{t} \approx 73\%$.

The optical intensity inside the sample I_s is calculated from the vacuum intensity by taking into account the surface reflection by Fresnel equations and the dielectric function of the material. With the index of refraction of germanium at the central wavelength of the pulse, $n(790\text{ nm}) = 4.7$, the intensity inside the sample can be written as[13]

$$I_s = n\left(\frac{2}{1+n}\right)^2 I_{vac} \approx 0.6 I_{vac}. \tag{15}$$

Supplementary Note 9: Orbital character of the CB and VB states

The orbital character for the CB and VB is depicted in the main text, Fig. 1b. Extensive hybridization of the atomic orbitals in the solid band structure leads to most of the conduction band having some percentage of 4*p* character, even in regions such as the Γ and L CB valley that are primarily of 4*s* character. In the valence band, the heavy- and light- hole bands are of similar energy, except where they bifurcate in the X valley. The split-off band is lower in energy and has more 4*s* character. Previous reflection spectroscopy measurements have identified the structure of the $M_{4,5}$-edge at 30 eV as pertaining to the Δ valley[14], but direct excitation to the Δ valley would require two near infrared photons. While such processes are indeed possible at the intensities used in the experiment, the cross-section for two-photon processes is considerably lower. A calculation of the projected density of states reveals that the Γ and L valleys are as much as 30% 4*p* character. These regions are thus visible in the core-level spectroscopy experiment, but it can be expected that near the band edge the sensitivity is decreased. This could explain why in Fig. 3a, near the assignment of the L_1 valley, the measured signal, although not zero, is weaker than expected.

Supplementary Note 10: Details on the first principle calculations

Real-time TDDFT as implemented in the real-space grid based Ab-initio Real-time Electron Dynamics simulator (ARTED)[15] code is used to simulate the excited-state carrier distribution created in the valence

and conduction bands by interaction with a few fs, 780 nm pulse with a peak intensity of 1×10^{11} Wcm^{-2} and the time profile shown in Supplementary Fig. 7a.

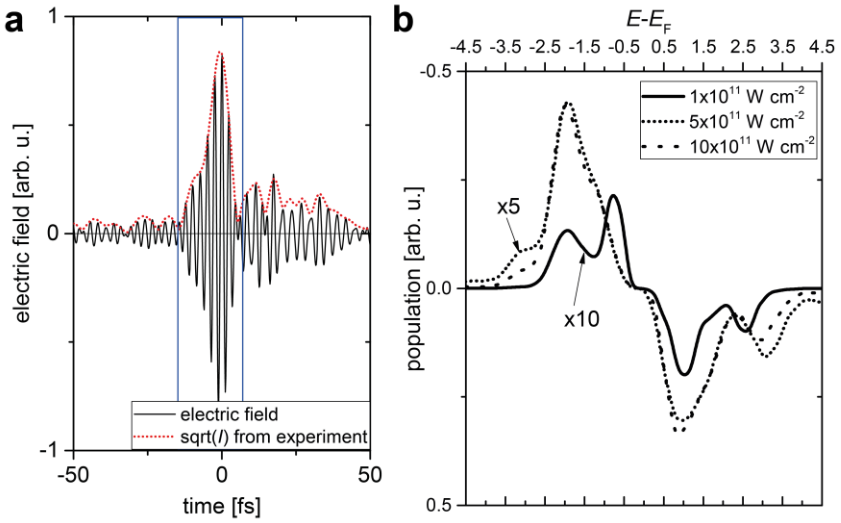

Supplementary Figure 7: *Electric field used for calculations modelling the visible-to-near infrared excitation pulse used in the experiment and time-dependent density functional theory (TDDFT) for different intensities. (a) The envelope of the pulse was measured by dispersion scan technique (dotted red line). The electric field is derived using the carrier-frequency of the pulse corresponding to a central wavelength of 780 nm (black solid line). For the calculations a time window of 20 fs width (blue rectangle) around the centre of the pulse was used. (b) Resulting populations calculated by TDDFT relative to the Fermi energy* E_F.

The temporal pulse profile is derived from experimentally measured intensity and spectral phase information using the dispersion scan technique[1] (cf. Supplementary Fig. 1b). The asymmetry of the electric field arises from a residual third order phase on the pulse. From the electric field in Supplementary Fig. 7a a 20 fs section around the peak electric field (blue rectangle in Supplementary Fig. 7a) was used for the calculations outlined here. Temporal windowing is necessary, since it is more computationally expensive to run longer segments and given the weak intensity outside the central segment it is not expected that significant population differences arise by excluding those. The real-time

TDDFT calculations are based on a 64 atom supercell of Germanium and employed norm-conserving pseudopotentials with 4 electrons in the valence. Wavefunctions are expanded on a 32x32x32 real-space grid and a uniform Γ-centred 8x8x8 grid is used for Brillouin-zone integration. The Tran-Blaha-Becke-Johnson potential is employed to describe exchange-correlation effects[16]. The subsequent occupation constrained eXcited-electron Core-Hole (XCH)[17] calculations are based on a 64 atom supercell of Ge, and employ a plane wave basis set with an energy cut-off of 40 Ry and the same 8 x 8 x 8 **k**-point grid as the TDDFT calculations. Exchange-correlation effects are described at the level of the Perdew-Burke-Ernzerhof[18] functional with a scissors correction employed to recover the full bandgap of germanium. Supplementary Fig. 7b shows the populations calculated by TDDFT for different intensities.

Supplementary References:

1. Silva, F. *et al.* Simultaneous compression, characterization and phase stabilization of GW-level 14 cycle VIS-NIR femtosecond pulses using a single dispersion-scan setup. *Opt. Express* **22,** 10181 (2014).

2. Timmers, H. *et al.* Polarization-assisted amplitude gating as a route to tunable, high-contrast attosecond pulses. *Optica* **3,** 707 (2016).

3. Madden, R. P., Ederer, D. L. & Codling, K. Resonances in the Photo-ionization Continuum of Ar i (20-150 eV). *Phys. Rev.* **177,** 136–151 (1969).

4. Codling, K., Madden, R. P. & Ederer, D. L. Resonances in the Photo-Ionization Continuum of Ne I (20-150 eV). *Phys. Rev.* **155,** 26–37 (1967).

5. Wang, H. *et al.* Attosecond Time-Resolved Autoionization of Argon. 143002–143006 (2010).

6. Bernhardt, B. *et al.* High-spectral-resolution attosecond absorption spectroscopy of autoionization in xenon. *Phys. Rev. A* **89,** 023408 (2014).

7. Herrmann, J. *et al.* Multiphoton transitions for delay-zero calibration in attosecond spectroscopy. *New J. Phys.* **17,** 013007 (2015).

8. Cao, R., Yang, X., Terry, J. & Pianetta, P. Core-level shifts of the Ge(100)-(2x1) surface and their origins. *Phys. Rev. B* **45,** 13749–13752 (1992).

9. Viña, L., Logothetidis, S. & Cardona, M. Temperature dependence of the dielectric function of germanium. *Phys. Rev. B* **30,** 1979–1991 (1984).

10. Zhan, T. *et al.* Thermal conductivity of sputtered amorphous Ge films. *AIP Adv.* **4,** 027126 (2014).

11. Bennett, B. R., Soref, R. A. & Alamo, J. A. D. Carrier-induced change in refractive index of InP, GaAs and InGaAsP. *IEEE J. Quantum Electron.* **26,** 113–122 (1990).

12. Kittel, C. *Introduction to solid state physics*. (Wiley, 1976).

13. Attwood, D. T. *Soft x-rays and extreme ultraviolet radiation: principles and applications*. (Cambridge Univ. Press, 2007).

14. Taniguchi, M. *et al.* Core-level reflectance spectroscopy of germanium by means of synchrotron radiation. *Solid State Commun.* **44,** 85–88 (1982).

15. Hirokawa, Y., Boku, T., Sato, S. A. & Yabana, K. Electron Dynamics Simulation with Time-Dependent Density Functional Theory on Large Scale Symmetric Mode Xeon Phi Cluster. in *2016 IEEE International Parallel and Distributed Processing Symposium Workshops (IPDPSW)* 1202–1211 (2016). doi:10.1109/IPDPSW.2016.200

16. Koller, D., Tran, F. & Blaha, P. Improving the modified Becke-Johnson exchange potential. *Phys. Rev. B* **85,** 155109 (2012).

17. Prendergast, D. & Galli, G. X-Ray Absorption Spectra of Water from First Principles Calculations. *Phys. Rev. Lett.* **96,** 215502 (2006).

18. Perdew, J. P., Burke, K. & Ernzerhof, M. Generalized Gradient Approximation Made Simple. *Phys. Rev. Lett.* **77,** 3865–3868 (1996).
